# Recent Innovation of Metal-Organic Frameworks for Carbon Dioxide Photocatalytic Reduction

**DOI:** 10.3390/polym11122090

**Published:** 2019-12-13

**Authors:** Alemayehu Kidanemariam, Jiwon Lee, Juhyun Park

**Affiliations:** School of Chemical Engineering and Materials Science, Institute of Energy-Converting Soft Materials, Chung-Ang University, Seoul 06974, Korea; alemayehu.kim@gmail.com (A.K.); dlwldnjstnd@naver.com (J.L.)

**Keywords:** metal-organic frameworks, composite MOFs, photosensitizer integrated MOFs, heterostructure quantum dot-MOFs assembly, metal ion doping, heterogeneous photocatalyst, CO_2_ transformation, CO_2_ reduction

## Abstract

The accumulation of carbon dioxide (CO_2_) pollutants in the atmosphere begets global warming, forcing us to face tangible catastrophes worldwide. Environmental affability, affordability, and efficient CO_2_ metamorphotic capacity are critical factors for photocatalysts; metal-organic frameworks (MOFs) are one of the best candidates. MOFs, as hybrid organic ligand and inorganic nodal metal with tailorable morphological texture and adaptable electronic structure, are contemporary artificial photocatalysts. The semiconducting nature and porous topology of MOFs, respectively, assists with photogenerated multi-exciton injection and adsorption of substrate proximate to void cavities, thereby converting CO_2_. The vitality of the employment of MOFs in CO_2_ photolytic reaction has emerged from the fact that they are not only an inherently eco-friendly weapon for pollutant extermination, but also a potential tool for alleviating foreseeable fuel crises. The excellent synergistic interaction between the central metal and organic linker allows decisive implementation for the design, integration, and application of the catalytic bundle. In this review, we presented recent MOF headway focusing on reports of the last three years, exhaustively categorized based on central metal-type, and novel discussion, from material preparation to photocatalytic, simulated performance recordings of respective as-synthesized materials. The selective CO_2_ reduction capacities into syngas or formate of standalone or composite MOFs with definite photocatalytic reaction conditions was considered and compared.

## 1. Introduction

Planet Earth is warming, and it is widely recognized that the global average surface temperature has increased considerably in the past few decades, entailing global warming [1] and devastation of entire ecosystems [2]; sea ice melting and rising ocean levels, shifting precipitation patterns, and migration of wildlife habitats are noticeable impact of global warming [3,4,5]. Ever since the discovery of fossil fuels in 1597 by Andreas Libavius [6,7], burning fossil fuel has been the primary means of energy exploitation, which in turn, aggravates the accumulation of greenhouse gasses (GHGs) in the atmosphere [8,9]. The effect of heat-trapping GHGs on the atmosphere was pictured by alteration of the Earth’s natural solar-irradiation-reflection cycle, leading to an environmental temperature surge [10,11]. 

**Figure 1:**
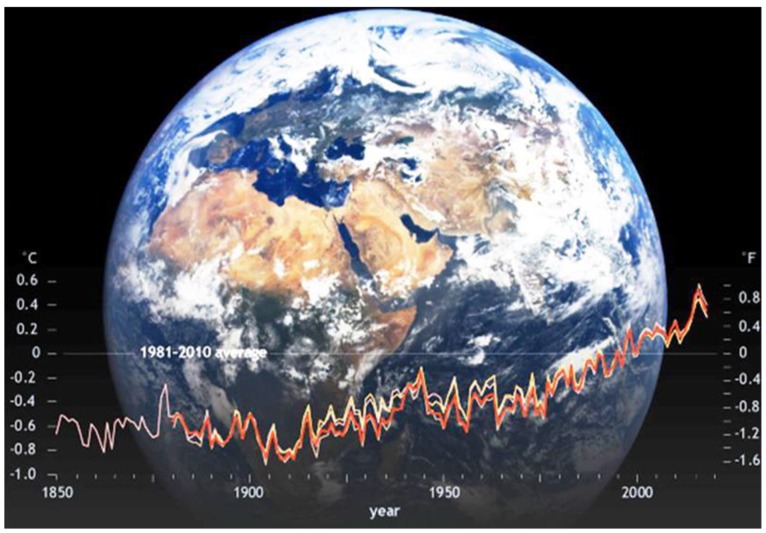
Yearly average surface temperature comparison (1850–2018). The background is an image of Earth from National Oceanic and Atmospheric Administration (NOAA)’s DSCOVR satellite (August 3, 2018). The average surface temperature for 1981–2010 is taken from four analysis groups, i.e., NOAA (red), National Aeronautics and Space Administration (NASA) (tan), the University of East Anglia (pink), and the Japan Meteorological Agency (orange). Adopted from the State of Climate in 2018 report from the American Meteorological Society (AMS) with permission [21]. Copyright 2018 NOAA.

The atmospheric carbon dioxide (CO_2_) concentrations in the preindustrial era and 2018 were 280 ppm and 407.4 ppm [12,13,14], respectively, implying that the accumulation of CO_2_ has increased throughout the industrialized era. The Intergovernmental Panel on Climate Change declares that, of all GHG constituents, CO_2_ emissions are the primary cause of climate change [15,16]. Data for 1951 recorded by the Goddard Institute for Space Studies indicated that global warming was indeed on course; the global temperature has risen by at least 0.8 °C by that time [17,18,19,20], and emissions have never ceased to date. According to the State of Climate 2018 report, due to an undisciplined boom of atmospheric GHG emissions, the average global surface temperature increased by 0.3–0.4 °C from 1981–2010 (Figure 1) [21]. Despite the endorsement of industrialized countries in favor of CO_2_ reduction by at least 5.2% over a decade ago (Kyoto Protocol) by attempting to employ green energy sources (e.g., solar, hydropower, and wind) for the gradual eradication of fossil fuel usage [22], implementation was impeded due to shortcomings of renewable energy utilization (i.e., affordability, accessibility, technological advancement, adequacy, and efficiency) [23,24,25,26]. 

On that basis, countermeasures for post-fossil fuel energy systems, such as physical capture of CO_2_ and storage (i.e., carbon capture and storage, CCS) [27] and/or reprocessing (carbon capture and utilization, CCU), have been formulated [28]. As for geological sequestration for CCS, different methods have been invoked at the emission source point of the huge plants (e.g., coal- and gas-fired industries) to reduce anthropogenic CO_2_ emissions; these include amine scrubbing, carbonation-calcination, and oxy-fuel [29] processes. Typical concentrations of flue gas from fossil-fuel-burning power plants released at nearly 1 atmospheric pressure are 15–16% CO_2_, 73–77% N_2_, 5–7% H_2_O, 3–4% O_2_, and a small amount of acidic gas [30]. In the amine scrubbing method, liquid amine compounds are used on a large scale as CO_2_ sorbent [31]; of all aqueous solutions, monoethanolamine (MEA), diethanolamine (DEA), and methyl diethanolamine (MDEA) have proven to be effective tools in capturing CO_2_ emissions at the source [32,33,34]. However, limitations in using these amine adsorbents include (I) mandatory pre-adoption stream purification (i.e., of SO_x_ and NO_x_) due to amine reactivity, [35,36,37], (II) corrosive liquid amine resulting in equipment wear-out during the adsorption process and the prevalent loss of sorbent [38], and (III) the excessive amount of regeneration energy required for sorbent recycling [39]. 

Another technology used to capture CO_2_ is carbonation-calcination, by which fixation consummated through carbonation reaction produces limestone (calcium carbonate, CaCO_3_) from quicklime (calcium oxide, CaO) and CO_2_, and then back-treating CaCO_3_ in a calcination reactor performs the reverse reaction (products will be CaO and CO_2_) [40,41,42]. Even though this method has a comparative advantage by overriding the pre-desulfurization process (unlike in amine scrubbing) [43,44], excessive heat of the carbonation (650 °C) and calcination (900 °C) procedures imposes a hefty economic impact [45,46]. CCS, being energy-intensive (for proper functioning and adsorbent regeneration), having poor stability, and in need of large-scale confined geographical storage reservoirs, has limited applicability [47,48]. 

Given that meeting the global energy demands in the foreseeable future relies on fossil fuel utilization, scientists around the globe have endeavored to acquire a quick remedy to alleviate atmospheric pollution not only by minimizing CO_2_ accretion but also conversion into valuable fuel products to meet foreseeable energy crunch [49,50]. Not long ago, many types of porous organic polymers, such as a conjugated microporous polymer, hyper-crosslinked polymers, polymer of intrinsic micro-porosity, covalent organic frameworks, covalent triazene frameworks, and porous aromatic frameworks, were notable as materials with good gas absorbance, storage, and catalytic compatibility [51,52,53,54,55,56,57,58,59,60]. Although the possession of compatibility morphological structures make porous organic frameworks feasible for use in gas CO_2_ storage [61,62], the advance utilization of this material faces obstruction due to low rates of gas diffusion-transport emanated from its lower density [63]. On the other hand, deployment of inorganic semiconductors, such as SrNb_2_O_6_, ZnGeO_4_, HNb_3_O_8_, Fe_2_V_4_O_13_, and BiWO_6_, for photoinduced CO_2_ reduction operation also encounters limitations emergent from their large band gaps and poor visible light collection. Moreover, absurd, non-porous structural characteristics of these semiconductors impose photocatalytic reactions to be carried on the external surface, resulting in bulk charge recombination [64,65,66]. This scenario significantly lessens the efficiency of the photocatalytic reduction process, and comparatively porous organic-inorganic frameworks are justified to pacify these setbacks [67,68,69].

## 2. Big Picture of Metal-Organic Frameworks (MOFs)-Photoinduced CO_2_ Metamorphosis

MOFs are products of repeatedly engineered amalgam (organic/inorganic) compounds in different regular dimensions (i.e., 1D, 2D, 3D) to yield coordinated porous polymers with organic ligands (linkers) and nodal metal ions as a cluster [70,71,72]. Organic linkers are created from organic material seeking enhanced CO_2_ adsorption and smooth photogenerated exciton drift to the reduction metal site [73,74,75,76], while metal clusters are reduction sites of the adsorbed CO_2_ compatible with excited electron and hole interaction. Metal ions in MOFs metallic clusters are usually divalent and trivalent metal ions of 3d transition metal, 3p metal, and/or lanthanides [77,78,79]. MOFs are still remarkable research topics for water splitting, organo-synthesis [80], gas absorption/storage-separation, drug delivery, and catalysis [75,77,81,82].

This material plays an indispensable role in CO_2_ photocatalytic reduction by virtue of its large gas-adsorption capacity and electronic collaboration, which originates from its enriched void morphological texture, chemical tunability, highly available porous reaction surface area, and densely coordinated unsaturated metal sites [83,84,85]. Although some materials have limited practical applications of CO_2_ absorption‒reduction directly at the source point of power plants due to their susceptibility to harsh conditions (e.g., acidity, alkalinity, heat, humidity), they are categorized as cost-effective with potent CO_2_ absorption and elimination ability [86,87]. Moreover, this material could be packed at the nanoscale to be deployed for atmospheric CO_2_ trapping and conversion to retrieve the environment being [88,89,90,91,92]. 

In addition, they can be designed from various types of high-density open metal sites and Lewis basic sites, which achieve great synergic interaction between CO_2_ substrate and pore site, rendering efficient CO_2_ absorption capacity [93,94]. Therefore, the construction of MOFs with the structural integrity to withstand harsh conditions (acid, alkaline, moisture, and heat) would be triumphant in tackling atmospheric CO_2_ pollution once and for all [95]. Diverse MOFs materials have been assembled from the different organic framework and nodal metal; selection of photocatalytic packs has accounted for some determinant aspects. Organ-metal compatible communication, higher CO_2_-adhesive void energy, and photolytic environment endurance are mentionable [96,97,98].

### 2.1. Factors Affecting Sorbent Material Selection

MOFs can be applied for gas adsorption, separation, storage, catalysis, and electronics [99,100]. The gas adsorption ability of this material could be improved by increasing the surface area, texture, and pores volume, while the catalytic activity could be improved by increasing the number of metal sites and adsorption enthalpy [101]. Logical scrutiny of the preferentiality of a given MOF depends on the palatability of the electronic and physical-chemical architecture for the photolytic redox reaction [102,103]. Therefore, the design and fabrication of MOFs equipped with superior CO_2_ uptake and redox potential for robust photocatalytic conversion is the ultimate ambition in the environmental pollution reduction arena, with pivotal elements of harmonious organo-metal cooperation, higher CO_2_ adhesive void energy, and photolytic versatility [96,97,98,104]. 

### 2.2. Electronic Structure and Photosensitivity of MOFs for CO_2_ Photolytic Reaction 

The photoreduction of CO_2_ with MOFs is accomplished by combining an array of proton-reinforced exciton-driven reactions with the oxidation of H_2_O to O_2_. To achieve this, the proper bandgap structure of MOFs is fundamental for the visible light radiation harvest and subsequent photolytic CO_2_ metamorphotic reaction [105,106,107]. The light-harvesting semiconductive nature of this material is crucial in converting incoming irradiation photons into activation energy to decrease the bandgap barrier. Upon incident irradiation, the generation of excitons is enacted from the valence bands (VBs) by leaving holes behind and drifting to active catalytic metal sites through conductive organic linkers [108]. Once the electron arrives on the metal center, it instantly activates and transforms the adsorbed substrate (CO_2_) to the corresponding intermediate product depending on the number of excitons and activation potential (Table 1). Photoinduced excitons complete travel in a truncated distance from an organic linker to a neighboring metal cluster, i.e., linker‒to‒metal cluster charge transfer (LCCT) [109], for subsequent reduction. It is worth noting that such materials can serve as photocatalysts or photocatalytic hosts in the photoreduction process. Heavier ligands create energy-level alignment flaws and asymmetry between constituent component orbitals (ligands and metal clusters) of MOFs, which forces electrons to transfer only short-range distances; as a result, some MOFs exhibit low electrical conductivity, which, in turn, causes loss of sensitivity towards visible light illumination [110]. However, many productive studies have been done to develop photoactive MOFs, capable of efficient synergetic communication within organic linkers and metal clusters, by augmenting semiconductivity dynamism [111,112]. 

### 2.3. CO_2_ Saturation Capacity

The other significant factor affecting MOF selection is uptake capacity; thus, the morphological properties of a photocatalytic material are directly related to the accessible surface area, porosity, number of metal sites, apt binding energy, and aromaticity [113,114]. A porous sorbent with chemically improved adsorption enthalpy is favored to trap void wall-encircling gases upon contact [115,116], and the void opening of the material affects its efficiency and applicability. In 2019, Sun et al. reported a carbonaceous biomass-based sorbent prepared from the receptacle and stalk of sunflowers spongy-like flesh through pyrolysis; this material was found to possess an abundant small-sized (<0.7 nm) porous surface area (nearly 3072 m^2^·g^−1^), with a high micropore ratio (79%), and intriguing structural suitability for pore wall-CO_2_ synergy, leading to greater substrate adsorption [117].

## 3. Photocatalytic CO_2_ Reduction Process Steps

**Figure 2:**
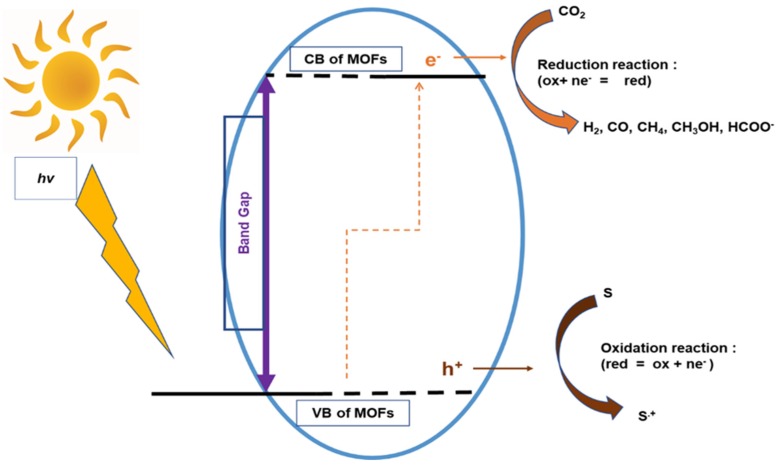
Diagrammatic representation of HOMO/LUMO band of exciton interaction over MOF photocatalyst for CO_2_ conversion upon irradiation (S = substrate, S^·+^ = oxidized substrate). HOMO: highly occupied molecular orbital; LUMO: lowest unoccupied molecular orbital; MOF: metal-organic framework; VB: valance bands; CB: conduction band.

As substantial eco-friendly CO_2_ transformer tools to mitigate environmental damage, MOFs are excellent photocatalysts with compelling energy utilization of bountiful solar energy and reduction capacity, mimicking the green plant photosynthesis route [118]. To better achieve the utilization of solar radiation for activation energy in CO_2_ reduction into valuable products, MOFs should possess suitable electronic structures and apt morphological properties. MOF-driven photocatalytic CO_2_ reduction processes have three major steps. (I) Light-harvesting: by design, photocatalysts are meant to collect much solar irradiation; for there to be adequate solar energy consumed by the photocatalytic reaction. Of the total solar irradiation, visible light contributes 42.3% (400 < λ < 700 nm), infrared radiation 49.4% (700 nm < λ < 1 mm), and ultraviolet (UV) rays 8%(λ < 400 nm) [119], implying that photocatalysts functioning under visible light are more favorable to have a higher energy-collection degree of freedom. (II) Separation of electron-hole pairs (charge assimilation): solar irradiation causes the excitation of valence electrons (leaving holes behind) from the VB or highiest occupied molecular orbital (HOMO) to the conduction band (CB) or lowest unoccupied molecular orbital (LUMO) [120,121], which are then separated and transported to the photocatalyst surface where the CO_2_ reduction is carried out (Figure 2). The charge recombination in semiconductors results in a decline in photocatalytic efficiency. Two methods have been devised to mitigate this problem: metal corroboration (surface metalation), in which metals serve as electron sinks for effective electron-hole separation and adopting heterojunctions [122], which achieves effective solar irradiation energy consumption. In addition, active catalytic surface exposure to solar radiation aggravates CO_2_ reduction adjacent to active sites [123]. (III) CO_2_-selective adsorption and conversion is the last step, in which the structural-driven thermodynamically stable CO_2_ substrate mandate the installation of highly efficient photocatalytic substances to achieve an appropriate endothermic selective redox reaction. Therefore, MOFs should inherently retain the semiconductivity feature with a lower bandgap and higher adsorption, and have an abundant activation unit within their structure. To convert CO_2_ to the subsequent formate, contrastively, MOFs’ CB potential should be more negative than the respective intermediate formation potential, so that excitons upon incident irradiation will easily drift to the metal center for instantaneous activation [124]. What’s more, the type of central metal significantly affects the performance of the photolytic process. This depends on how well it interacts with the substrate activation concomitantly due to morphology or porosity (high pore volume to surface area ratio) of the MOF, and binding energy will alter the activity; the binding energy (void surface and substrate interaction) needs to be high for CO_2_ selective reduction [125,126]. For further fostering solar irradiation harvesting, MOFs may be coupled with photosensitizers (PSs). PSs are illumination-sensitive materials that have compatible band edge alignment to MOFs’ structure with the lowest possible CB to assist hot multi-exciton injection to active catalytic sites [127,128]. On the other hand, materials will be incorporated into the morphological texture of MOFs to advance the physical adsorption capacity of the void crew. From an environmental mitigation perspective, MOFs should be durable as well as exhibit strong reduction performance [129,130].

## 4. Photocatalyst MOFs Category 

Given their outstanding CO_2_ chemisorption and activation performance, MOFs have been an important area of research for GHG pollutant reductions. It is the target of all researchers to develop MOFs with exellnet CO_2_ photoreduction efficiency, and this effort escalates aspect-wise innovation. Thus, depending on the focus area, findings concerning MOFs could be divided into two main groups: the advancement of (I) the electronic structure and (II) morphological properties [131]. The electronic structure implies the semiconducting nature of the material; this feature is responsible for light irradiation harvesting and photoinduced multi-exciton generation-injection to the catalytic active central metal node, thereby achieving CO_2_ activation [132]. Equivalently, the morphological texture of the assembly significantly affects the reduction performance; a material having a porous structure with higher CO_2_ binding energy and apt metal cluster has superiority for selective substrate reduction [133]. Overall, the smooth interaction between the organic linker and nodal metal plays an important role in the advancement of photolytic reduction.

Recently, countless breakthroughs have been accomplished regarding MOFs, targeting innate synergistic activity intensification. However, those reported publications are not reviewed yet. The objective of our work was to briefly document and unveil the latest progress from respective motivation through material fabrication to application in a clear and concise manner with technical reflection. We aimed at providing better awareness of MOFs present prospects toward CO_2_ reduction.

### 4.1. Zr-Based MOFs

Zirconium (Zr)-based MOFs are known for their unusual structural suitability for photo-driven catalytic reaction, in which Zr(IV)-carboxylate coordination bonds enable the synthesis of MOFs with high surface area, better electronic structure, and reaction environment flexibility. Furthermore, the vulnerability of Zr-based MOFs to the functionalization of different moieties via many synthesis methods is an exemplification of their superiority [134,135]. Accordingly, Zr-based MOFs draw much attention in the exploration in photocatalyst input. 

In 2017, Tu et al. studied the photocatalytic activity improvement of a Zr-based MOF (UiO-66) by microwave-assisted post-synthesis for incorporating a cation (titanium), with some Zr(IV) metal in Zr-Oxo clusters of UiO-66 replacement by Ti(IV) to produce UiO-66 (Zr/Ti)-M [136]. The Brunauer-Emmett-Teller (BET) surface area of this MOF before and after Ti metal incorporation was measured, and the value indicated minor-deviation [969.7 m^2^·g^−1^ of UiO-66, and 965.8 m^2^·g^−1^ of UiO-66 (Zr/Ti)-M] due to the proper pore occupancy. On the other hand, the bandgaps of UiO-66 and UiO-66 (Zr/Ti)-M were 4.00 and 3.75 eV, respectively. The HOMO and LUMO of UiO-66 were 3.40 and −0.60 V (vs. NHE), respectively, while UiO-66 (Zr/Ti)-M showed a HOMO of 3.45 eV and a LUMO of −0.30 eV (vs. NHE). As mentioned above, the replacement of some Zr metal with Ti indeed improved the electrical property of the MOF through band-edge alignment of Ti to the MOF template. The photocatalytic activity enhancement of UiO-66 (Zr/Ti)-M is due to the upgraded interfacial charge transfer of the exciton from the linker Benzene-1,4-dicarboxylic acid (BDC) to the Zr-oxo cluster, and the donation of the electron for Zr(IV) (to be an electron sink) is achieved through Ti(IV) introduction [137]. This n-type semiconductor has proven to have potential as a photocatalyst for CO_2_ photoreduction. The metalation of MOF on its core structure will advance the solar responsiveness by enhancing the photocatalytic reduction reaction through prolonged multi photoinduced exciton generation and transmission to the active site, resulting in adsorbed CO_2_ activation [138]. Aside from photocatalytic reduction performance, the metalation of Zr-based MOF shows resilient performance in a harsh reaction environment. 

Moreover, in 2018, Chen et al. discovered a Zr-based MOF with extraordinarily great pH resilience (hyperstability of all Zr-MOFs) to harsh (HCl, pH = 1 and concentrated NaOH) reaction condition [139]. Via a top-down synthesis process, MOFs (Zr_2_C_44_H_22_O_12_N_4_ (ZrPP-1)) with NbO-like topology, featuring immense Zr(IV)-oxo chains linked by polyphenolate groups on four peripheries of eclipse-organized porphyrin macrocycles, were successfully constructed by deploying non-intercrossed Zr(IV)-pyrogallic porphyrin MOFs, ZrPP-1. These possessed the stability even under severely alkaline conditions with an exquisite photocatalytic CO_2_ deoxygenation reduction reaction (i.e., CO_2_ to CO). Compared to other Zr(IV)-carboxylates, this MOF [140,141,142,143,144] has maintained its excellent status. From a N_2_ adsorption-desorption isotherm, a porous ZrPP-1 MOF had a Langmuir surface area of 512.5 m^2^·g^−1^ (BET, 744.5 m^2^·g^−1^). Taking advantage of its high alkaline reaction condition resistance, different metals were incorporated into the organic ligand of this MOF (ZrPP-1-M, where M = H_2_, Fe, Co, Cu, Zn), of which co-integrated n-type photocatalyst MOFs exhibited superb photoreduction performance when collaborating with a proton-donating agent. The defined and well-ordered active metal centers, apt bandgap (1.78 eV) and metalation (Co) at the porphyrin core were responsible for the superior CO_2_ uptake at 273 K (87.7 cm^3^·g^−1^) and selective photocatalytic reduction (Table 2). The efficient photoinduced exciton separation and transfer of this cobalt-based MOF were due to its narrow bandgap and the apparently crossed Fermi levels of the ZrPP-1 entity. Furthermore, this MOF could be recovered and recycled for three rounds without obvious loss of morphological integrity and performance.

**Figure 3:**
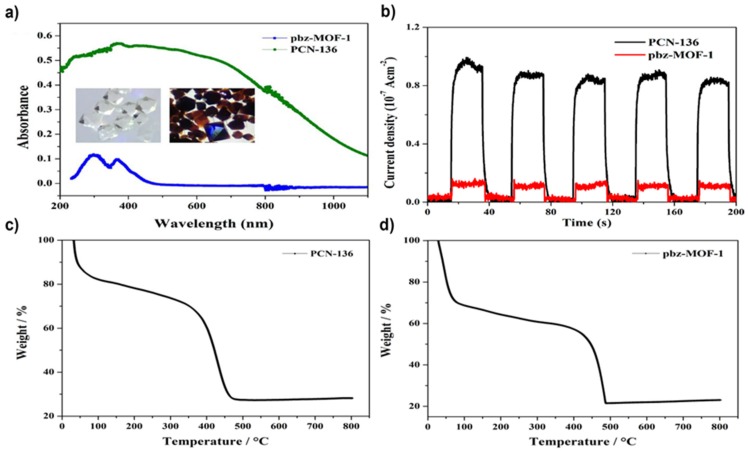
(**a**) UV−vis spectra of pbz-MOF-1 and PCN-136; insets are the optical pictures of pbz-MOF-1 (left) and PCN-136 (right), (**b**) Transient photocurrent responses of pbz-MOF-1 and PCN-136 in 0.5 M Na_2_SO_4_ aqueous solution under visible-light irradiation. The thermogravimetric analysis (TGA) curves from room temperature to 800 °C at the heating rate of 10 °C·min^−1^ of (**c**) PCN-136 and (**d**) pbz-MOF-1. Reproduced with permission [140]. Copyright 2019 American Chemical Society.

The structural agreement with the electronic property of MOF stack promotes efficient charge transfer with enhanced solar irradiation collection, even though MOFs have the same metal cluster, framework topology, particle size, crystallinity, and pore size arrangement; thus, it is plausible that MOFs may experience different CO_2_ photocatalytic reduction potentials rendered from structural deviation [179]. Recently, structural modification of MOFs has been studied for enhanced photolytic exertion, achieved by alteration of MOFs structure via large π-conjugated ligand inclusion onto Zr-based MOF (Zr_6_(μ_3_-O)_4_(μ_3_-OH)_4_(OH)_6_(H_2_O)_6_(HCHC), where HCHC = hexakis (4-carboxyphenyl) hexabenzocoronene), denoted as PCN-136. In the fabrication process, a solvothermal reaction was used to synthesize pbz-MOF-1 (Zr_6_(μ_3_-O)_6_(μ_3_-OH)_2_(Ac)_5_(OH)(H_2_O)(HCBB)) from Zr^4+^ salt and hexakis(4′-carboxy[1,1′-biphenyl]-4-yl) benzene (HCBB) ligand followed by oxidative cyclodehydrogenation under FeCl_3_ catalyst. Thereby, PCN-136 was constructed by post-synthetic annulation of pbz-MOF-1 via transforming the hexaphenylbenzene core of the HCBB ligand to the planarized hexabenzocoronene ligand (HCHC). Semiconductor characteristics of PCN-136 were assessed to have 1.18 eV as its optical bandgap, while LUMO and HOMO are −0.75 and 0.43 V (vs. NHE), respectively; the lower LUMO value from the reduction potential of CO_2_ to formate guarantees the photoreduction potential of this MOF catalyst. Furthermore, PCN-136 CO_2_ uptake capacity at 273 K was found to be 61.01 cm^3^·g^−1^. This suggests that communication between sp^2^-bonded carbon atoms within hexabenzocoronene fragments is indeed responsible for the amplified CO_2_ adsorption capacity of PCN-136. Photocatalytic CO_2_ reduction potential of PCN-136 was shown by Qin et al. to have selective CO_2_ reduction to HCOO^−^, yielding 10.52 µmol formate, which is 3-fold more than the pristine pbz-MOF-1 conversion yield in the same reaction condition (Table 2). This reduction outperformance is attributed to extended π-conjugated hexabenzocoronene-based linkers in PCN-136, which will assist its photoinduced redox reaction by broadening the visible light spectrum absorption range and increasing current density (Figure 3a,b). Thermogravimetric analysis (TGA) reveals that the as-prepared material exhibits better thermal resistance relative to the precursor complex (Figure 3c,d) [140]. 

**Scheme 1:**
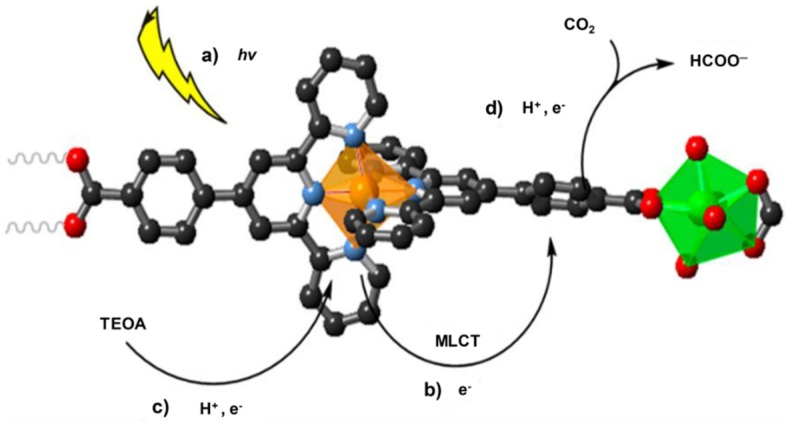
The illustration of Zr-based MOF (AUBM-4) photolytic CO_2_ reduction mechanism upon light illumination, (**a**) Incident irradiation, (**b**) Exciton generation and transmission of Ru-to-cptpy MLCT, (**c**) Ru-reducing via protonation from TEOA sacrificial agent, (**d**) cptpy^•−^ radical will provide electron to neighboring CO_2_ molecule for conversion. Color coding (excluding hydrogen): Ru(gold), Zr(green), C(gray), O(red), N(blue). Reproduced with permission [145]. Copyright 2019 American Chemical Society.

The incorporation of material that has high light-harvesting ability and surplus electron density to Zr-based MOF will provide extraordinary CO_2_ reduction capacity of the templet, and the inclusion of Ru-complex in Zr-based MOFs advances the photoinduced reduction and conversion not only by supplying higher oxidation-reduction capacity but also long-lived exciton generation with better charge carrier separation [180,181]. An exemplary Zr-based MOF (AUBM-4, American University of Beirut Materials) embellished with Ru-based ligand has been reported for improved photolytic operation, the catalyst assembly achieved through (Ru(cptpy)_2_), [bis(4′-(4-carboxyphenyl)-terpyridine)Ru(II)complex] embodiment on the backbone of ZrO_8_ cluster [145]. AUBM-4 is a one-dimensional structured MOF BET with a surface area of 50 m^2^·g^−1^ and 1.88 eV optical bandgap; this absorption band is supplied from ruthenium metallic ligand (inside MOF architecture) of singlet metal-to-ligand charge transfer (MLCT). The further demonstration of AUBM-4 electronic structural activity in response to light irradiation for CO_2_ photoreduction (Scheme 1) is believed to have Zr-O metal cluster with 4d orbital Zr metal and Ru(cptpy)_2_ supportive organic ligand, indicating that the ruthenium metal center is responsible for light-harvesting and exciton provision to organic linker cptpy (4′-(4-carboxyphenyl)terpyridine). This organic linker will form radical cptpy^•−^, and then there is a direct reduction of CO_2_ via Zr-metal coordination; notably, the presence of electron donors is vital to enhance the repeatability of the reduction process by protonating deprotonated Ru-metal center. As reported by Elcheikh Mahmoud et al., AUBM-4′s superb CO_2_-to-HCOO^−^ conversion capacity was in the same reaction condition compared to other catalysts, such as PCN-222 [Zr_6_(µ_3_-OH)_8_(OH)_8_-(TCPP)_2_], NH_2_-UiO-66(Zr), 253-Ru(5,5′-dcbpy)(CO)_2_Cl_2_, MIL-101(Fe) [Fe_3_F(H_2_O)_2_O(BDC)_3_], Zr-SDCA-NH_2_, NNU-28, Ir-CP, and Eu-Ru(phen)_3_-MOF (Table 2) [146,147,148,149,150,151,152,153].

**Scheme 2:**
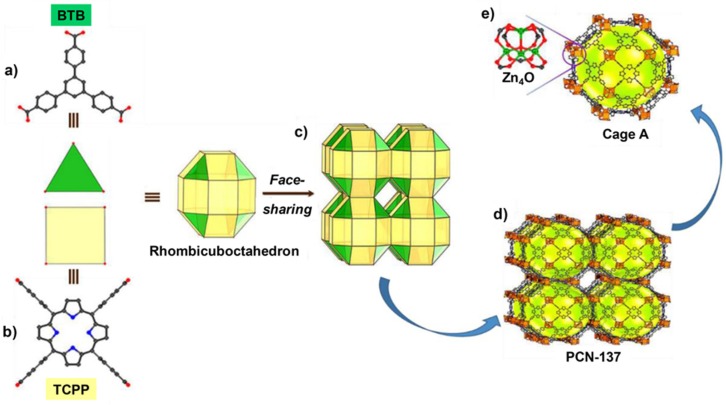
Structure of PNC-137; (**a**) BTB, (**b**) TCPP, (**c**) 3D structure formation, (**d**) (3,4,7)-connected topology of PNC-137, (**e**) 7-connected Zn_4_O cluster on each cage. Reproduced and modified with permission [163]. Copyright 2019 American Chemical Society.

**Scheme 3:**
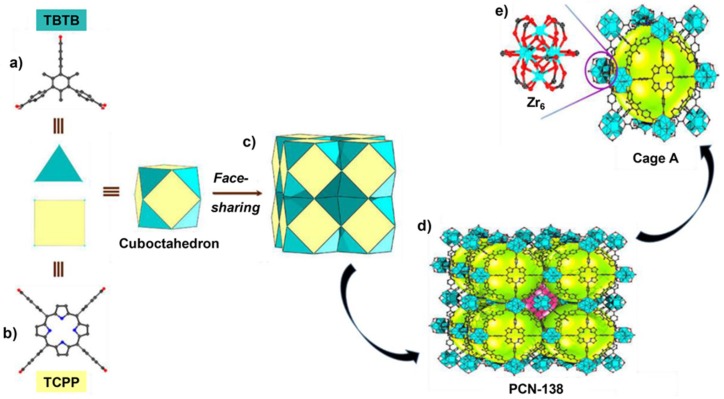
Structure of PCN-138; (**a**) TBTB, (**b**) TCPP, (**c**) 3D structure formation, (**d**) (3,4,12)-connected topology of PCN-138, (**e**) 12-connected Zr_6_ cluster. Reproduced and modified with permission [163]. Copyright 2019 American Chemical Society.

To maximize the photocatalytic conversion ability of Zr-based MOF 2D metal-organic layers (MOLs), crystalline is a perfect ally for the enhancement of light-harvesting capability of a MOF template. Characteristics like separated ligand by metal connected nodes, weak chromophore coupling region, and efficient energy transfer to reaction site attribute for 2D MOLs, mirroring the natural photocatalytic reaction approach of green plants [182,183]. Using this material in combination with Zr-based MOFs for photocatalytic CO_2_ conversion would result in better energy collection and utilization, thereby boosting photocatalytic conversion. In 2019, Hu et al. developed 2D light-harvesting MOF analogs [i.e., metal-organic-layer (MOL)], which were efficient multiple photo-exciton injectors in short duration and transport to Re- and Ir-based reaction metal site for photocatalytic reduction of CO_2_ to CO and/or HCOOH [184]. Therefore, the incorporation of MOLs catalytic antenna enhances the directional multi-photon-generated electron injection and improves transport to the reductive site of the MOF catalytic center (Re- and Ir-based), affecting prohibited charge recombination to increase the photocatalytic performance of the respective conjugated MOFs. In another study, the integration of ligands with distinguished engineered solids proved to be an effective avenue for the designability of MOFs equipped with advanced CO_2_ uptake and photoreduction; this MOF entwined bond formation of explicit building units (molecules and clusters) with an extended skeleton through topological consideration [185]. To further elaborate the above-mentioned stipulation, Qiu et al. recently contrived two MOFs-mixed ligands with two 3D Archimedean solids (Ass), rhombicuboctahedron and cuboctahedron. Despite the complication of ASs design [i.e., (I) ASs have identical apex, (II) at least two different regular polygons should constitute to each member of ASs, (III) each regular polygon in ASs (e.g., square, pentagon, hexagon, octagon, decagon, equilateral triangle) should have equal edge-length], these ASs are potential candidates to synthesize endured MOFs with careful design and manipulation. The number of metal nodes to be used affects the construction of ASs-based MOFs, commonly 7-connected Zn_4_O clusters and 12-connected Zr_6_ clusters. The two ASs-based MOFs were constructed based on a polyhedron-blueprint from stacking of rhombicuboctahedron (PCN-137) and cuboctahedron (PCN-138); it is worth noting that 1,3,5-benzene(tris)-benzoate (BTB) and/or 4,4′,4″-(2,4,6-trimethylbenzene-1,3,5-triyl)tribenzoate (TBTB) organic ligands and tetrakis(4-carboxyphenyl)-porphyrin (TCPP) ligand had equilateral triangles and square, respectively (Scheme 2a,b). This geometrical side-length equivalency asserted the utilization of ASs to construct rhombicuboctahedron cast (3,4,7)-connected topology PCN-137 via face-sharing (Scheme 2c,d). Onto the rhombicuboctahedron cast, the μ_4_-oxygen atom at the equator was connected to two Zn1 and two Zn2 atoms, fashioning a Zn_4_(μ_4_-O) cluster (Scheme 2e). Contrarily, 12-connected Zr_6_ metal nodes were attached to four discrete TCPP ligands in the central plane through four carboxylate moieties and eight TBTB ligands at the top and bottom of the central plane via eight carboxylate moieties (Scheme 3a,b), producing a PCN-138 framework using two different-sized cages (Scheme 3c,d) with interconnection of three or four Zr_6_ clusters to deprotonated TBTB or TCPP ligand, respectively (Scheme 3e). The nitrogen sorption analysis showed that the BET surface area of PCN-138 was 1261 m^2^·g^−1^, and its CO_2_ uptake capacities was 63.07 and 40.72 cm^3^·g^−1^ at 273 and 298 K, respectively. This n-type semiconductor MOF (PCN-138) had VB(HOMO) 0.96 V vs. NHE and CB (LUMO) of −0.86 V vs. NHE. Thus, the more negative value of PCN-138 LUMO from the CO_2_ to formate reduction potential (−0.28 V vs. NHE) certified that PCN-138 was viable for CO_2_ reduction potential (20.21 µmol formate) [163]. 

### 4.2. Zn-Based MOFs 

Although extensive research has been done on the utilization of noble metal and dye molecules as PS to achieve enhanced photocatalytic performance with selective product reduction of CO_2_, and their application is seemingly impractical due to lack of abundance and stability issue under light irradiation [186,187,188,189], the inclusion of noble-metal-free co-catalyst together with sensitizer possessing high light absorption ability and irradiation stability is the best way to counteract the above-mentioned problem. Moreover, a zinc (Zn)-based MOF could be concocted in a modified dimensional structure for the ramification of better adsorption-reduction capacity. It is noteworthy that the integration of improved ligand-to-metal synergy achieves better photothermal resilience; this aspect not only increases the recyclability of ordinary MOFs’ photocatalysts but also critical determinant characteristics for PS design [190]. To elucidate this aspect, in 2018, Ye et al. studied ultrathin two-dimensional Zn-MOF [Zn porphyrin], based on TCPP ligand, nanosheet with BET surface area of 445 m^2^·g^−1^ and CO_2_ adsorption potential of 103.8 cm^3^·g^−1^. This Zn-MOF was applied as a PS in cobalt complex co-catalyst ZIF-67 [Co_2_(OH)L](ClO_4_)_3_; (L = N[(CH_2_)_2_NHCH_2_(m-C_6_H_4_)CH_2_NH(CH_2_)]_3_N) in a MOF/ZIF CO_2_ photocatalytic reduction system [154]. Photoreduction was carried out by feeding pure CO_2_ (99.999%) to the preassembled bulk Zn-MOF and ZIF-67 (0.18 µmol), yielding 21.20 µmol of CO and trace H_2_ (91% selectivity of CO over H_2_) (Table 2). 

Having higher BET surface area, ultrathin (4.7 nm thickness) Zn-MOF semiconducting PSs possess admirable electronic structure, attributing enhanced irradiation harvest and thus the increased photolytic performance of MOF/ZIF hybrid system achieved for the selective deoxygenated reaction of CO_2_ under mild redox condition. The hybrid photocatalyst outperforms bulk MOF, affirming the synergistic communication between the matrices and sensitizer. This MOF shows better thermal stability, and TGA confirms that decomposition of TCPP causes loss of mass and pores to subsequent decline of photoreduction efficiency after 400 °C. Therefore, incorporation of a suitable semiconductor as a PS to a MOF complex will aggravate the photocatalytic sensibility for CO_2_ reduction with detracted photon radiation degradation; thus, making noble metal obsolete in the design and synthesis of photoreductant MOFs is another accomplishment. 

Heating MOFs’ nanomaterial can result in the fabrication of metal oxide nanostructures. Effective selection and controlling of the synthesis route of nanostructure oxide formation yield dynamic photocatalysts, which even outperforms the pristine components for CO_2_ reduction [191,192,193]. When it comes to the employment of semiconductor nanostructures as photocatalysts, enlarged absorption band, better charge-carrier separation, and high surface area to volume ratio are attainable. The effect on the performance of hybridized MOFs at various temperatures is discernable. For example, Yan et al. recently reported an investigation targeted on procuring affordable (low-cost), environmentally friendly, and efficient photocatalyst of ZnMn_2_O_4_. This work has been secured in novel porous self-assembly microsphere photocatalyst with controlled particle size, large specific available surface area, many reactive sites, acknowledged redox reaction ability, harsh condition resilience, and apt bandgap [155]. 

ZnMn_2_O_4_ nanoparticles (NPs) were constructed from calcinated powder of zinc acetate and manganese acetate; on the other hand, ZnMn_2_-ptcda (ZMO, where ptcda = perylene-3,4,9,10-tetracarboxylic dianhydride) MOF precursor was calcinated at four different temperatures (350, 450, 550, and 650 °C) to obtain powders denoted as ZMO-350, ZMO-450, ZMO-550, and ZMO-650, respectively. Flower-like ZMO-based microspheres exhibited an increase in particle size with the inclination of calcination temperature. The BET surface areas of ZMO-350, ZMO-450, ZMO-550, and ZMO-650 were 24.7, 45.8, 109.1, and 8.4 m^2^·g^−1^, respectively. Unlike the smaller surface area drawback of ZMO-650, its electronic structure was appreciable due to the lowest bandgap (2.15 eV) [194,195], while the precursor ZnMn_2_O_4_ NP had smaller BET surface area (2.7 m^2^·g^−1^) and large bandgap (2.1–2.6 eV); the large bandgap for the small ultrafine particle-sized nanostructure (ZnMn_2_O_4_) was due to the quantum confinement effect. Due to increased photo harvest and hindered charge recombination, the ZnMn_2_O_4_ microspheres possessed better CO_2_–to-CO conversion photoreduction activity than ZnMn_2_O_4_ nanostructure (Table 2), rendered from electro-structural and morphological disparity.

Although physic-thermal treatment promotes morphological and electronic characteristics of MOFs, the type of organic ligand and heat tolerance still engender repressed applicability. As a mitigation strategy, the envelopment of NPs in the hosting MOFs matrix seems productive [196]. More recently, Han et al. addressed enhanced photocatalysts via incorporation of NPs functionality in MOFs. The assembled material was fabricated by direct encapsulation of core-shell (Au@Pd) NPs in Zn-based MOF (MOF-74) catalytic material, where an Au@Pd core-shell (i.e., Au NPs used as the core to grow Pd near-spherical shell) produced Au@Pd@MOF-74 MOF showed the complete conversion of CO_2_ to CO [159]. Furthermore, the inclusion of Pt NPs in pure MOF and composite Pt/MOF-74 and Pt@ Au@Pd@MOF-74, respectively, enhanced the CO_2_ reduction, for both methanation and reverse water-gas shift (RWGS), to CO at low concentration. Due to simple morphological controllability, the MOF-74 catalyst was selected to be used as the template to envelop NPs. Resulting from weak photon adsorption potential standalone, the Au@Pd NPs and Au@Pd@MOF-74 experienced lower CO_2_ reduction [197]. Furthermore, the higher Pt loading and surface exposure assist charge trapping, and the enhanced synergistic interaction between Pt and Au@Pd core-shell structure boosts the overall reduction potential of the composite catalyst with acceptable stability. 

It is noteworthy that the speculated capacity of Au@Pd@MOF-74 is to photoreduce CO_2_ to CO, while Pt/MOF-74 has a capacity of converting CO_2_ to CH_4_. Therefore, Pt/Au@Pd@MOF-74 is expected to possess the cumulative effect of those two co-catalytic materials, and potential candidates for CO_2_ reduction to both CO and CH_4_; when this material is used in photocatalytic reduction reaction, CO_2_ would be converted to CH_4_ (with 84% selectivity) and 100% CO generation when employed in RWGS reaction. 

### 4.3. Ti/TiO_2_-Based MOFs

Titanium dioxide (TiO_2_) semiconductor’s optical absorption is confined to only UVspectrum as a result of its large bandgap 3.0–3.2 eV possession, and hence sensitivity to a low portion of total solar irradiation (UV) and prevailing charge-recombination, both of which diminish the photocatalytic performance of titanium semiconductor [198]. In another perspective, the handy-designability of the TiO_2_ nanotube array structure allows enhanced light absorption spectrum band and higher charge-carrier separation [199,200]. In 2018, Li et al. reported a light-concentrating photoreactor to be used in CO_2_ photocatalytic reduction driven by inorganic semiconductor catalyst (TiO_2_ nanotube array) prepared by anodization method capable of harvesting incident irradiation. The BET surface area of this catalyst was 26 m^2^·g^−1^, the photoinduced redox reaction was conducted between pure CO_2_ and water on the catalyst, and capacity of CO_2_ to CH_4_ conversion was increased from 0.7 to 20.67 µmol·g^−1^·h^−1^. This was attributed to the enhancement of incident light intensity and surface oxidation state property of the catalyst [201].

TiO_2_ material could be molded in discrete methods, the results of each being positive with respect to specific electro-physic-chemical aspects. It is customary for the incorporation of noble metal (for example, Pt, Pd, Rh, Ru, Au, Cu, Cu, Pt, ZnPd, and AuCu) into bare TiO_2_-based photocatalyst in order to suppress possible charge recombination of the electron-hole by providing a separation heterojunction, and asserting better-photogenerated exciton transmission to the active catalytic metal site (where the reduction takes place) [202,203,204,205]. The utilization of a noble metal for photocatalyst design shortens the practical application emanated from a lack of abundance and stability [206]. However, the annealing method avoids the need for noble metal is avoided. Reports from Kar et al. affirmed the development of standalone TiO_2_ photocatalyst by flame annulation of TiO_2_ nanotubes (FANTs) for efficient CO_2_ photocatalytic reduction without any noble metal co-catalyst [207]. Solo TiO_2_ photocatalysts were proposed as stable photocatalysts based on FANTs converting CO_2_ into CH_4_ under visible light irradiation with a high formation rate of 156.5 µmol·g^−1^·h^−1^. The promoted CO_2_ photocatalytic performance of FANTs stemmed from the sole rutile crystalline existence, increased visible light absorption, and square morphology.

### 4.4. Fe-Based MOFs

Iron (Fe)-based photocatalysts are the most typical in the photocatalytic reduction arena; Fe-oxide semiconductors have tremendous performance in photon collection and subsequent reduction of substrates proximate to the active exciton electron site. The ability to harvest visible light for multiple hot-electron injections, alterable structure, and material abundance (cost-effectiveness) is a major factor for researchers to focus on Fe-based photocatalyst development. In particular, the integrability of Fe-oxide with other materials with enhanced structural communication between the constituents broadens the innovation of perovskite and MOFs for CO_2_ reduction [208]. Lately, Liu et al. developed a MOF (Ag/AgCl@ MIL-53-Fe) with improved visible-light photocatalytic activity via solvothermal integration of plasmonic Ag/AgCl NP and MIL-53-Fe. Due to the enhanced synergistic photoinduced charge transfer of Z-scheme morphological arrangement between the components in the composite MOF (Ag/AgCl@MIL-53-Fe), the as-prepared composite photocatalytic material exhibited higher solar-driven reduction potential compared to the precursor pristine material [209], and the bandgap of 2.64 V showed the possibility for further study in pollutant reduction.

Furthermore, environmentally adaptable MOFs’ photocatalysts could be designed using Fe-O. Dao et al. proclaimed three solvent-free MOFs for photocatalytic reduction of CO_2_ with the motif of boosting photoconversion efficiency and selectivity of CO evolution. Remarkably their result forbade any evolution of H_2_, CH_4_, and/ or liquid product (i.e., HCOOH). This corroborated that all Fe MOFs have extraordinary selective photocatalytic reduction of CO_2_ toward CO; thus, three Fe-based MOFs were experimentally synthesized by hydro-solvothermal reaction and examined for CO_2_ deoxygenation photoreduction activity: NH_2_-MIL-53(Fe) [Fe(OH) -(NH_2_-BDC)]·G, NH_2_-MIL-88B(Fe)[Fe_3_O(H_2_O)_3_(NH_2_-BDC)_3_]Cl·G, and NH_2_-MIL-101(Fe) [Fe_3_O(H_2_O)_3_(NH_2_-BDC)_3_]Cl·G [144]. 

This solvent-free MOF for photoreduction contains TEOA as a sacrificial electron donor; due to the presence of an amine functional group in the photocatalyst, light absorption and CO_2_ reduction affinity at the gas–solid interface will be upgraded [210]. Compared to the usual solvated CO_2_ photoreduction, this solvent-free MOF affords extra surface area of contact for the redox reaction and is environmentally adaptable. All Fe-based MOFs have higher LUMO values to that of CO_2_ to CO reduction potential (−0.53 V vs. NHE), insinuating achievable CO_2_ photoreduction [211]. Of the three Fe MOFs, NH_2_-MIL-101(Fe) has superior CO_2_ photoreduction capacity and is plausible for CO_2_ deoxygenating redox reaction, with a conversion capacity of 87.6 µmol·g^−1^, which is nearly 5.6 times higher than NH_2_-MIL-53(Fe) (Table 2). 

The efficient performance of NH_2_-MIL-101(Fe) emanates from its better electrical dynamism. Conductivity from Nyquist plots of NH_2_-MIL-101(Fe) is 4.1 × 10^−6^ S·cm^−1^, while that of NH_2_-MIL-53(Fe) is 1.1 × 10^−6^ S·cm^−1^ [212]; this deviation arises from their peculiar framework structures. However, since all these photocatalysts have been designed to operate in a solvent-free system, their performance in the presence of moisture, acid, and contaminants is not clearly stipulated, and it is noteworthy that this hostile condition resilience of photocatalyst is critical to specify the application area of CO_2_ reduction [213].

### 4.5. Ni-Based MOF

Logically, metal nodes supply concentrated catalytic activation sites in MOFs, signifying the compatible selection and designability of metal clusters propelling the photoreduction process by cherishing unsaturated coordination of metal sites into the framework. For instance, the incorporation of nickel (Ni) metal ion in the MOFs as metal clusters will not only increase the photolytic conversion purified gases but also dilute forms of CO_2_ [214,215]. Recently, Han et al. studied nodal metal dependence of MOF-based CO_2_ photocatalytic reduction using Ni-based MOF in monolayers [ultrathin Ni MOLs] for efficient photocatalytic reduction of diluted CO_2_ (10%), which were constructed by hydrothermally ultrasonication process and examined [161]. This material had a 48.9 m^2^·g^−1^ BET surface area. The photocatalytic conversion was carried out in a photoreactor containing a photocatalyst, Ni MOLs, and [Ru(bpy)_3_]Cl_2_·6H_2_O (bpy = 2,2′-bipyridine) as PS and TEOA (triethanolamine) as a sacrificial agent under visible light irradiation; CO and H_2_ were main throughputs of the redox reaction, and Ni incorporation possessed superior performance compared to Co analogy (Table 2). Collating Ni ion with the conjugated ligand with enlarged coordination is believed to advance the synergy, and thereby the catalytic performance. In 2018, Zhu et al. revealed a 2D conductive MOF nanosheet with co-catalyst Ni_3_(HITP)_2_, (HITP = 2,3,6,7,10,11-hexaiminotriphenylene) for CO_2_ photoreduction potential under visible-light irradiation fortified by [Ru(bpy)_3_]^2+^, PS, and TEOA as sacrificial agent [164]. Ni_3_(HITP)_2_ is a π-stacked stratified MOF with extended π-conjugated coordination, showing superb conductivity comparable to graphene (5000 S·m^−1^), which in turn enables Ni_3_(HITP)_2_ to be an electron sink reservoir of photogenerated excitons sourced from PS [216,217]. 

**Figure 4:**
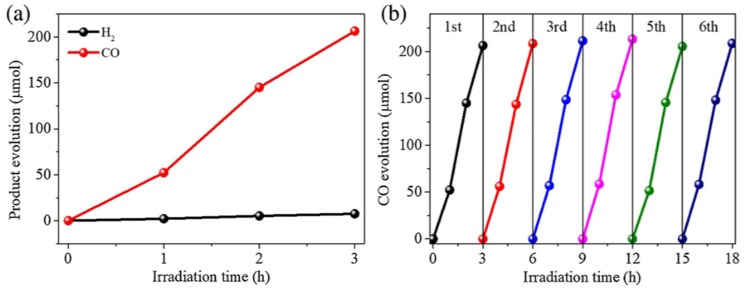
(**a**) Ni_3_(HITP)_2_ CO and H_2_ evolution vs. illumination time capacity, containing Ru(bpy)_3_Cl_2_·6H_2_O under 80 kPa and CO_2_ atmosphere at 4 °C, **b**) The recyclability tests of Ni_3_(HITP)_2_ under the same experimental conditions. Reproduced with permission [164]. Copyright 2018 Elsevier.

In addition, the elevated exposed surface area (up to 630 m^2^·g^−1^) of Ni_3_(HITP)_2_ is responsible for the provision of extra catalytic reaction sites, which will aggravate the CO_2_ reduction potential of hybrid MOF [218]. This MOF has been confirmed to persist its original photocatalytic performance for over six subsequent cycles with enhanced selectivity of CO production over H_2_ (Figure 4a,b). The CO_2_ to CO conversion performance is greatly influenced by the concentration of co-catalyst and sensitizer. The physical structural assessment of Ni_3_(HITP)_2_ has been confirmed to have a hexagonal phase. 

### 4.6. Cu-Based MOF 

Copper (Cu) clustered MOFs are capacitated with remarkable CO_2_ absorption potential with excellent selectivity over N_2_ at ambient conditions, even under stiffened reaction conditions in large amounts as the simplest path. Moreover, Cu clustered MOFs can be regenerated and reused with original adsorption capability [219]. The incorporation of metal ions into the MOFs will increase the photocatalytic performance of CO_2_ reduction to respective products, rendered from the enhanced photogenerated charge carrier and separation with apt minimum decay; thus, carefully engineered Cu-based MOFs are effective in long-lived electron generation and substrate reduction. Goyal et al. provided an orthorhombic structured NP of MOF from Copper (II)/Zeolitic Imidazolate Framework-8 (2Cu/ZIF-8N2) photocatalyst in ammonium hydroxide concentration via hydrothermal process used for CO_2_ conversion to methanol; the as-synthesized MOF had a bandgap of 1.75 eV, VB (0.72 V vs. NHE), and CB (−1.02 V vs. NHE @ pH = 7), implying lower VB than that of ZIF-8 (2.41 V vs. NHE), meaning the oxidation reaction took place in ZIF-8 [220]. On the other hand, the more negative-value CB of synthesized MOF to the CO_2_ to MeOH potential (−0.38 V) affirmed this MOF’s reduction potential with apropos metal loading. 

The electronic configuration and electronic sensibility confirmed that Cu metal is a strong candidate in heterojunction formation to desensitize charge recombination. In 2019, Hu et al. reflected affordable three-component heterojunction photocatalyst to convert CO_2_ to CO without an organic sacrificial agent. This heterojunction was constructed from Cu_2__-x_S nanotubes laminated with a carbon layer (C-Cu_2-x_S) and g-C_3_N_4_, and the ternary heterojunction was C-Cu_2__-x_S@g-C_3_N_4_ [168]. The precursors of Cu-containing material in this ternary heterojunction MOF is HKUST-1 MOF, with the periodic distribution of Cu nodes and carbon-rich organic ligand attributes for the candidacy of HKUST-1. Via implementation of apt sulfidation and calcination, C-coated Cu_2-x_S (C-Cu_2__-x_S) tubes with the hollow structure are collected, followed by mixing of C-Cu_2__-x_S with g-C_3_N_4_ under the hydrothermal condition to acquire C-Cu_2__-x_S@g-C_3_N_4_ [221,222]. Many techniques have been used to characterize the topology, morphology, stability, and performance. In the heterojunction, different ternary MOFs have been fabricated by alternating Cu (C-Cu_2__-x_S) weight ratios; among these, 0.71 wt.% loading has shown superb photocatalytic reduction of CO_2_ to CO (evolution rate of 88.5 µmol·h^−1^·g^−1^), with a suppressed competitive H_2_ evolution. This composite MOF has promoted 97.6% CO selectivity (Table 2). Therefore, this ternary composite MOF has outperformance in photocatalytic activity, and being environmentally friendly and low-cost, this MOF is feasible to be employed in the photoreduction of CO_2_ to CO [223]. 

**Figure 5:**
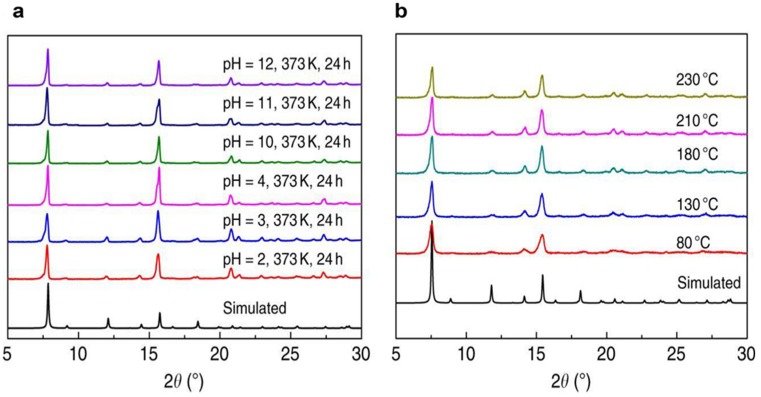
FJI-H14 harsh condition stability. (**a**) PXRD in different acid/base environments under boiling water and (**b**) temperature-dependent PXRD patterns. Reproduced with permission [219]. Copyright 2017 Nature Communications.

Meager resilience of MOFs towards heat, humidity, and acidic condition obstructs their application in coal- and gas-fired plants on emissions source points. For mitigating the aforesaid limitation, MOFs need to be carefully designed with greater flexibility. Cu, as a metal cluster could be used to pave this aspect. Liang et al. reported a design of rod-shaped blue crystal from the dense open metal site (OMS) and Lewis base site (LBS) of Cu(II)-MOF (FJI-H14) [219]. The as-synthesized Cu-clustered MOFs possessed efficient CO_2_ adsorption tendency under the humid and acidic conditions in the large amounts. Fundamentally, FJI-H14 could be regenerated and reused with original adsorption capability with distinguished acidic endurance. Moreover, powder x-ray diffraction (PXRD) confirmed that these MOFs operated at a high temperature, showing intact stability, underwater, and 2 ≤ pH ≤ 12 (basic and acidic media) (Figure 5a,b). This is all attributed to higher synergy between the Cu central metal and BTTA linker. 

### 4.7. Composite MOFs

Most MOFs exhibit low chemical stability and poor reaction environment (such as alkalinity, acidity, and heat) resilience; this stringent trait limits the advanced applicability on CO_2_ emission-spot of huge power plant [224,225]. The incorporation of functionalization material to MOFs is intended to mitigate the performance restraints of both components (i.e., MOFs template and moieties), showing that the hybrid material performs better than the standalone constituents. The selection of functionalization components depends on the compatibility of band edge and morphological aspect to each other, prevailing efficient photocatalytic performance [226,227,228,229]. Generic advantages of MOFs in the composite are accessible porous surface area, uniformly distributed crystalline, and structural maneuverability, while merits of various functionalization materials are peculiar optical, magnetic, electrical, and catalytic properties, escorting MOFs with proper functional moieties. Thereby, the novel physic-chemical property will be obtained from this sophisticated composite material, affirming a comprehensive combined effect for photocatalytic performance [230,231,232,233]. 

#### 4.7.1. Ti/TiO_2_ Composite

**Figure 6:**
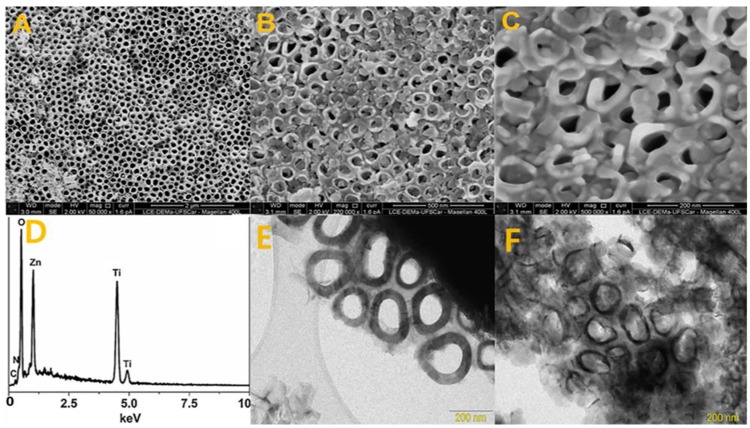
Morphological structure of TiO_2_NT pre- and post-assembly with ZIF-8; (**A**) FEG/SEM image of pure TiO_2_ nanotubes, (**B**,**C**) FEG/SEM images of NPs of ZIF-8 incorporated into Ti/TiO_2_NT, at two different magnifications, (**D**) SEM-EDS spectrum of Ti/TiO2NT-ZIF-8, (**E**) TEM of Ti/TiO_2_ nanotube, (**F**) TEM of Ti/TiO_2_NT-ZIF-8. Adopted and edited with permission [238]. Copyright 2007 Elsevier.

As mentioned above, titanium-based semiconductor for photocatalytic reaction has drawbacks due to poor visible light absorption spectrum range and significant charge recombination. TiO_2_ has merits of being non-toxic, abundant/naturally occurring (in forms of rutile, anatase, and ilmenite), structurally adaptable, and having a colored structural texture [234]. In 2015, Anitha et al. claimed the development of n- and p-type transparent semiconductors from TiO_2_-based thin films and nanomaterials for photocatalytic activities in atmospheric pollutant reduction for their classical electrically conductive property. In addition, as-proposed titanium materials were feasible for synthesis, modification, and energy-related phenomenon [235]. It has been suggested that furnishing and doping MOF templates with this titanium material would result in a great outcome due to morphological rapport [236,237]. Cardoso et al. reported Ti/TiO_2_-based composite MOF for photocatalytic reduction of CO_2_ [238], and remarkable thermal and chemical resilience zeolitic imidazolate frameworks (ZIFs) are still important aspects of MOFs [239]. Porous ZIFs are composed of coordinated tetrahedral divalent cations (Zn^2+^, Co^2+^, and Cd^2+^) with a zeolite-like framework consisting of imidazole spacer ligands [240]. ZIF-8 (Zinc-based compound [Zn(mIM)_2_], mIM = 2-methylimidazole), taking advantage of its porous nature with a higher exposed surface area (1881 m^2^·g^−1^) and attractive stability, is famous for its utilization due to selective chemisorption, activation, and co-catalytic activity in CO_2_ photoreduction. Additionally, the presence of an imidazolate linker basic site assists the CO_2_ activation tendency of ZIF-8 [241]. Fabricated MOF material topological property has been scrutinized by different techniques. Scanning electron microscopy imaging reveals the original porous topology of TiO_2_ nanotube (NT) (Figure 6A); TiO_2_NT has dimensionally characterized nanotube with a diameter of 90 to 100 nm, the thickness of 20 to 30 nm, and length of nearly 1.2 µm, which is input for advancing gas adsorption capacity of the hybrid MOF. After ZIF-8 embodiment on TiO_2_NT, NPs with an average size of 50–57 nm agglomerate and are distributed over the entire surface of NT by nucleation and particle growth without clogging the initial nanotube voids (Figure 6B,C). The EDS pattern (Figure 6D) ensures the attendance of Ti, C, N, O, and Zn elements in the assembly complex. Moreover, TEM images (Figure 6E,F for Ti/TiO_2_NT and Ti/TiO_2_NT-ZIF-8, respectively) confirm the incorporation of ZIF-8 NPs in-between and within TiO_2_NT.

Due to Ti^4+^ structural-incapacitation to adsorb CO_2_, bare Ti/TiO_2_NT shows very low CO_2_ photoreduction to ethanol (EtOH) and methanol (MeOH), while utilization of Ti/TiO_2_NT-ZIF-8 photocatalyst results in 430-fold of EtOH and 20-fold of MeOH, proving exceptional effects of MOF complex toward CO_2_ reduction due to the hybridization of titanium-based nanotube and ZIF-8 decoration, affirming that EtOH is indeed favored. Therefore, Ti/TiO_2_NT-ZIF-8 photocatalyst acquires efficient CO_2_ adsorption and conversion to fuel, owning substantial breakthroughs in the composite MOFs arena [242].

Remarkably, porous ZIF-8 material is thermally stable up to 200 °C, and it can easily be fabricated by treating Zn(NO_3_)_2_·6H_2_O with excess mIM at room temperature. Notably, this gives a quick production of narrow-sized, regularly distributed nanocrystals [243]. This new subclass zeolitic-based MOF could be coupled with different semiconducting materials, yielding improved photocatalytic material [244,245].

**Figure 7:**
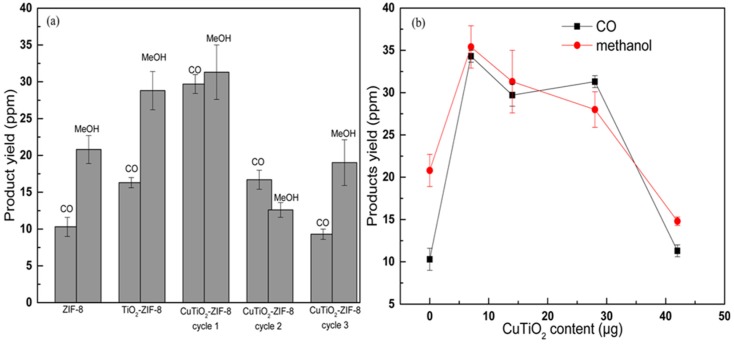
Contrastive illustration of ZIF-8 composite and pristine photolytic activity. (**a**) Effects of membrane composition on formation capacity and durability, (**b**) Effects of dopant NP concentration on product yield. Reproduced with permission [246]. Copyright 2017 American Chemical Society.

Maina et al. [246] reported that the same template MOF (ZIF-8) was infused with coordinated semiconductor NP (TiO_2_ and Cu-TiO_2_) via rapid thermal deposition (RTD) for CO_2_ photolytic activity. It was found that the CO_2_ reduction rate of this heterostructure was higher compared to the pure precursor (Figure 7a). Along with repeated cycles, the composite material exhibited lower efficiency persistently, especially for CO evolution rate; furthermore, the production yield varied depending on the concentration of inorganic dopant semiconductor NPs (Cu-TiO_2_) onto porous template (Figure 7b): 7 µg loading of Cu-TiO_2_ NP outperformed reduction potential of CO_2_, yielding 2170 ppm·g^−1^.cat. of CO and 2238 ppm·g^−1^.cat. MeOH. Therefore, the controlled assimilation of inorganic NPs within the MOF membrane not only increased the charge transportation (by hindering possible charge recombination) but also improved the photocatalytic reduction potential with better stability.

CO_2_ capture and conversion would be entertained by hiring TiO_2_ nanomaterial as a constituent for MOFs composite fabrication. The surveillance for TiO_2_ material for photocatalytic application as standalone or as a complex is due to its low cost, high chemical inertness, and adaptive structural texture [247,248].

Charge carrier density improvement is vital for enhanced CO_2_ reduction, and the interfacial synergy is a causative agent for this aspect. Therefore, theoretically, the morphological controlling of MOFs-bundle upon assembly is paramount to the catalytic activity; to sink this stipulation, a bifunctional nanocomposite material from integrated TiO_2_ nanosheet (NS) (BET surface area 42 m^2^·g^−1^) and NH_2_-UiO-66 (BET surface area 871 m^2^·g^−1^) [Zr_6_O_4_(OH)_4_(NH_2_-BDC)_6_, BDC = Benzene-1,4-dicarboxylic acid] MOF by forming heterojunction through in-between tight interaction, used not only to capture but also to photo-reduce CO_2_ under UV-vis illumination [166]. The segment-wise analysis has confirmed that due to the cumulative effect, the purpose of each consisting component is to advance the CO_2_ photocatalytic transmutation performance of the nanocomposite MOF. TiO_2_ and NH_2_-UiO-66, respectively, are chosen for their photocatalytic activity and adequate void surface with enhanced catalytic activity; therefore, the composite material (TiO_2_/NH_2_-UiO-66) possesses long-lived photoinduced excitons generation capacity and enhanced substrate adsorption for an efficient heterogeneous (gas–solid) CO_2_ photoreduction process [249]. 

In another study, controlled loading of TiO_2_ nanofiber onto the MOFs pattern was speculated to improve charge separation, while worsening electron-hole recombination. TiO_2_-based MOF (NH_2_-UiO-66) composite with enhanced charge transfer and superior CO_2_ adsorption-reduction [250] and different mass NH_2_-UiO-66 particles were coated on 1D titanate or anatase nanofiber. In the architecture component-integrating process, NH_2_-UiO-66 loading varied with range and with the highest being 33 wt.% titanate/MOF composite (t-300-MOF), while anatase/MOF composite (T-300-MOF) had 15 wt.% of NH_2_-UiO-66. Controlled morphologies of composite material have a paramount effect on its photocatalytic activity; for example, efficient charge separation owned by exciton short distance travel route, small-sized MOFs are more favored for light absorption [251]. Due to its regulated framework, T-300-MOF hybrid material has better durability [252].

In addition to controlling morphology, pre-conformance of incorporating material for their compatible communication has beneficial effects for bettering photocatalytic activity. Titanium oxide is known to have a large bandgap, which limits its practical application in visible light irradiation for light-absorbing semiconductor, signifying that the assimilation of titanium oxide to another material to mitigate this restraint, which would further advance the development of novel solar illumination hospitable complex. Given the above-stated advantage of achievable titanium oxide modification, such attempts in photocatalytic complex construction are viable [253,254,255]. 

He et al. discovered ternary composite MOFs for the collaborative conversion of CO_2_ to CO and CH_4_ from TiO_2_/Cu_2_O/Cu_3_(BTC)_2_ with a molar ratio of 20:0.55:0.45, respectively, under visible light irradiation [256]. The purpose of TiO_2_/Cu_2_O composites in MOFs is to grant better heterojunctions for appropriate charge separation and light absorption, while Cu_2_O and Cu_3_(BTC)_2_ (BTC = 1,3,5-benzenetricarboxylate) are used to provide electrons with the robust coordinative unsaturated metal (Cu) sites for the activation of adsorbed CO_2_ to subsequent conversion into intermediate and formate products upon light-illumination. On the other hand, generated holes will undergo water hydrolysis to form H^+^, O_2_, and OH radicals for the reaction with the CO_2_ intermediates to form syngas. 

Atomic layer deposition (ALD) of titanium-based semiconductor with other material improves the core/shell photocatalytic performance [257]. Zhao et al. reported nanocomposite-structure Au/Al_2_O_3_/TiO_2_ ALD of Al_2_O_3_ inside the interweaved layer of Au and TiO_2_, revealing plasmonic Au NP (NP) for CO_2_ photocatalytic reduction underwater by enhanced photoinduced charge separation with a hindered aspect of exciton-hole recombination [258,259]. More recently, Feng et al. reported improved (anatase-rutile) TiO_2_ hybridized MOF from MIL-125, followed by ultrathin MgO uniform overlayer deposition over porous TiO_2_ through ALD for CO_2_ photoreduction to CO; this surface modification assisted the deactivation of TiO_2_ surface state and prohibited surface charge recombination [260]. Porous TiO_2_ (PTiO_2_) is used to prepare the MIL-125 template by pyrolysis and calcination operation. Given its abundance, and the nontoxic and adaptive structure, MgO via ALD is feasible [261,262]. Of all composition, five layers of MgO (5ALD) is optimum configuration, showing better CO_2_ conversion to CO (54 µmol/g). In comparison, 5ALD assembly has 52 m^2^·g^−1^ BET surface area and 3.0 eV band gap; however, below (thinning) and further (thickening) will attribute charge recombination and insufficient exposed surface area for photoreaction, respectively, causing photocatalytic activity drop. The incorporation of MgO into TiO_2_ surface causes the production of Ti^3+^ [261,263], postdated by intimation with surface oxygen vacancy forming surface oxygen vacancy/Ti^3+^ (due to defective surface), which could advance CO_2_ adsorption and activation (conventional two- and eight-electron) by forming formats of intermediate product (Equation 1‒3) [249], implying facilitation of reduced output [264,265,266].

Ti^3+^ + CO_2_ → Ti^4+^ − CO_2_^−^ (1)

OH^−^ + CO_2_ → HCO_3_^−^ (2)

Ti^4+^ − O_2_^−^ + CO_2_ → Ti^4+^ − CO_3_^2−^ (3)

#### 4.7.2. CdS-Based Composite MOF 

As one of the inorganic compounds, CdS is a semiconductor with a promising bandgap of 2.43 eV responsive to visible light irradiation, and the conductivity of this material simultaneously increased with incident illumination intensity [267]. This material has an important role in photolytic reduction if crosslinked with other inorganic and/or MOF components. The design of integration depends on the interest of the ultimate goal [268]: for instance, one of many designs is chiral inorganic nanomaterial for their application in catalysis, optics, sensing, and biomedical applications [269,270]. CdS can be assembled with MOFs for auxiliary application and catalyst fabrication. In 2015, Kuang et al. reported the synthesis procedure for chiral CdS nanotubes composited to the MOF template for sensor applications [271]. The composition of CdS into MOF is a routine research activity with the aim of obtaining enhanced photocatalytic material. In 2012, Gao et al. reported the successful synthesis of graphene CdS composite for photocatalytic activity; the composite formation was confirmed for uniform distribution of CdS over graphene sheets through the solvothermal process, and the as-prepared material unveiled enhanced visible-light-driven charge generation and separation sufficient for the photocatalytic reaction [268]. 

CdS could be coupled with different MOFs to enhance the electrophysical property of the assembly. Zhao et al. developed an efficient CdS/NH_2_-UiO-66 hybrid membrane for CO_2_ photocatalytic material to alleviate the limitations of powder-form catalysts (i.e., light scattering, easy agglomeration, and tough recovery) [272]. The carrier material for the hybrid membrane was chitosan: low cost, proper gas permeability, and the presence of abundant amine getting along with carboxyl groups in chitosan compound makes it a better candidate for membrane matrix of the hybrid material because gas permeability and amine-, carboxyl- groups give it effective CO_2_ adsorption features [273]. The hybrid membrane exhibited enhanced CO_2_ photoreduction with better selectivity than the initial pure membrane components (i.e., CdS and NH_2_-UiO-66) under mild reaction conditions. 

The photocatalytic reduction performance of this material exhibits 99% CO selective reduction of CO_2_; this hybrid membrane’s MOF activity is exceptional compared to other ever reported research findings [153,274,275,276,277,278,279,280,281,282]. In addition, the incorporation of another semiconductor to the hybrid membrane increases photocatalytic photoinduced redox reaction by the prevalent facile exciton transfer with prohibited charge recombination, which is due to the provision of synergistic reaction between the MOF and semiconductor [137,283]. However, the ternary composite based on CdS theoretically possesses artificial heterojunction, which will passivate the bulk-charge recombination subsequent to excitation and simultaneous increase of photoelectron-generation, giving an efficient ternary composite MOF capable of CO_2_ photocatalytic reduction to CO with evolution rate of 235 µmol·g^−1^·h^−1^ and 85% selectivity over H_2_ (41 µmol·g^−1^·h^−1^). Composite MOF was constructed by incorporation of CdS and molecular redox catalyst through UiO-bpy MOF to yield CdS/UiO-bpy/Co, with UiO-bpy used as a linker for inorganic semiconductor and redox catalyst, as reported by Chen et al. [177]. 

Furthermore, CdS incorporation into MOFs with inherent surface area to void volume ratio improves photolytic performance. In a report by Ding et al., the CdS/MIL-101(Cr) composite photocatalyst was used as a visible-light-driven CO_2_ to CO convertor. The fabrication of this composite MOF was achieved through (I) MIL-101(Cr) construction via the hydrothermal process and (II) n-type semiconductor CdS encased within MIL-101(Cr) cavities via in-situ growth of the double solvent route [176]. 

#### 4.7.3. Zr-Based Composite MOF

Zr-based MOF with the usual hexanuclear Zr cluster linked by terephthalates [284] remains an attractive research field, owning high thermal resistance, chemical stability, and facile functionalization [285], granting for adsorption [286,287], and catalytic applications [288]. The combination of this material to other inorganic metals or semiconductors will theoretically improve the uptake and transformation of pollutants. In 2016, Baek et al. confirmed the activity of Cu crystalline on selective hydrogenation of CO_2_ to fuel, and this property was obtained by the encapsulation of Cu nanocrystals within Zr-based MOF [Cu⊂UiO-66]. This complex catalyst exhibited 100% selective production of methanol and an 8-fold greater yield compared to the benchmark catalyst (Cu/ZnO/Al_2_O_3_) [289]. The integration of conducting material and broadened light harvester is an efficient way of boosting the photocatalytic activity of porous Zr-based MOF. Meng et al. reported three-component Zr-based composite MOFs constituted from oxygen-defective ZnO(O-ZnO)/rGO(reduced graphene oxide)/UiO-66-NH_2_ with Z-scheme heterojunction via solvothermal operation, aiming to boost photocatalytic performance by photogenerated electron-hole separation [165]. 

Defecting ZnO with oxygen encourages extended visible light optical absorption edge [290]; thus, it is unfortunate that this oxygen defective semiconductor has limitations in effective charge separation, and hence, fast photogenerated charge recombination will be prevalent. The electronic interaction between both semiconductors (UiO-66-NH_2_ and O-ZnO) is attributable to the upgrade of visible-light absorption spectrum range of the ternary composite MOF, hence the advanced photocatalytic activity of the composite through the increased number of photogenerated electron/hole pairs. This ternary Z-scheme composite MOF has superb CO_2_ photocatalytic conversion to CH_3_OH and HCOOH, confirming that Z-scheme heterojunction inauguration plays important role for photolytic activity; moreover, the presence of oxygen vacancy in the ternary heterojunction photocatalyst improves catalytic performance (i.e., it is responsible for 2-fold CH_3_OH and 1.5-fold HCOOH reduction increment) [291].

Given that MOFs have the superb large porous surface area and tailorable morphology, they are still an indispensable field of study for pollutant reduction, especially in pursuing improved performance with an alliance with semiconducting material [292]. Quantum dots (QDs) are an astonishing material for their broader absorption spectrum, long-lived electron generation-assimilation, endured photostability, and large optical spectrum. Furthermore, their optical spectra adjustability via particle size alteration ranks QDs at the first semiconductor division for incoming irradiation responsiveness [293,294,295]. In 2013, Jin et al. developed a hybrid material of QDs and MOF for enhanced light harvesting, functionalization of porphyrin-based MOF with CdSe/ZnS core/shell QDs for enhancement of energy transfer from QDs to MOFs, and expanding the light absorption spectrum of visible light [296]. Given that CO_2_ is a thermodynamically stable carbon molecule, extensive activation energy is a pre-requisite to effect reduction [297]; numerous authors struggle to find a route for perovskite complex implementation in photocatalytic reaction by tackling their imminent limitation (i.e., poor moisture condition resilience), but perovskite’s shortcoming could be addressed via composting to MOFs [298,299]. Toward elucidating an efficient inorganic halide perovskite CsPbBr_3_ QDs/UiO-66(NH_2_) heterojunction-type nanocomposites MOF for photocatalytic CO_2_ conversion in non-aqueous media, CsPbBr_3_ QDs have been constructed from Cs-oleate solution into PbBr_2_ solution at high temperature, with a few reagents used in the synthesis process (i.e., Cs_2_CO_3_, PbBr_2_, octadecene, oleic acid, and oleylamine) [141]. On the other hand, UiO-66(NH_2_) has been fabricated from zirconium tetrachloride and 2-amino-1, 4-benzenedicarboxylic acid through the solvothermal process [285]. Conjugation of these two gives a heterojunction [CsPbBr_3_ QDs/UiO-66(NH_2_)] nanocomposite; remarkably, the main particles in CsPbBr_3_ QDs/UiO-66(NH_2_) nanocomposites possess special interaction with other particles by employing surface functionalization [300]. Given that electronegativity of the existing amino group in MOF (UiO-66(NH_2_)) will strongly attract and interact with electropositive crystalline perovskite (CsPbBr_3_ QDs), this in-between chemical synergy is responsible for CsPbBr_3_ QDs/UiO-66(NH_2_) nano junction hybrid structural integrity with enhanced electrochemical sensibility. The HOMO/LUMO of CsPbBr_3_ QDs and UiO-66(NH_2_) were determined as 2/−0.89 and 1.25/−1.02 eV [301,302], affirming the charge transfer from photoexcited exciton of CsPbBr_3_ QDs to UiO-66(NH_2_). This material showed a great reduction capacity of CO_2_ to CO and CH_4_, as reported by Wan et al. [166].

#### 4.7.4. Co-Based Composite MOF

Cobalt (Co), as an abundant element, tends to be doped with heteroatom carbon for a photolytic reaction. Because of the highest porous surface area for gas penetration, additional catalytic cavity creation due to porous carbon atom doping, and metal particles stably fixated on the carbon pattern, interweaving Co with MOFs entails robust photoreduction activity [303,304]. Co metal is important in directional charge transportation when coupled with suitable MOFs, selectively capturing and efficiently photoreducing CO_2_ under visible-light irradiation for syngas generation. The incorporation of a single Co atom into MOF encourages directional excited electron transfer from porphyrin (organic linker) to the reaction site Co (metal cluster) by significantly decreasing electron-hole recombination [167]. 

The integration of metal ions (i.e., Co and Zn) into the porphyrin moieties encourages the electron transfer and higher CO_2_ adsorption-reduction; syngas generation with this strategy is recommended as an effective means of CO_2_ reduction to mitigate fuel crises [305]. A report from Zhao et al. reported a MOF photocatalyst for CO_2_ photoreduction clustered with Co^II^ with efficient adsorption and conversion performance [156], naming it as the hexanuclear cobalt cluster-based MOF (Co_6_-MOF). This photocatalyst with [Ru(bpy)_3_]Cl_2_·6H_2_O (Ru-Complex) PS affected higher CO_2_ reduction to CO and H_2_. The opportunity of the modifiable electronic structure of Co asserts the possible integration with MOFs, yielding better component-wise communication favoring CO_2_ reduction. An ultra-small silver NP-doped Co-based MOF (Co-ZIF-9) via the photo-deposition route to yield Ag@Co-ZIF-9 has achieved enhanced CO_2_ to CO conversion under visible light irradiation [306]. The photocatalytic redox reaction has been conducted by treating 99% pure CO_2_ (1 bar) in a photoreactor pre-loaded with this catalyst and Ru-complex. The doped amount of Ag NPs in Co-ZIF-9 were proven to have a direct proportionality to the evolution rate of CO while decreasing the production of H_2_. Of all Ag NP deposition, 7.13 wt.% is found to be optimum, improving the multi-photogenerated exciton injection and separation for CO_2_ reduction sites. 

Interestingly, the composite formation of Co with perovskite would show higher uptake and metamorphotic performance. Recently, a composite photocatalytic catalyst has been developed by a combination of perovskite and MOF, fabricated via direct deposition of ZIF (Zn/Co-based zeolitic imidazolate framework) on the surface of CsPbBr_3_ quantum dots, for the attainment of a combined effect for efficient CO_2_ photoreduction [178]. The as-prepared composite hybrid structured CsPbBr_3_@ZIF possesses enhanced moisture resilience, CO_2_ adsorption, and charge separation-transmission. 

In 2019, Wang et al. designed a congregated photocatalyst from template zeolitic imidazolate framework-67 (ZIF-67) and Co_3_O_4_ of hollow multi-shelled structures (HoMSs) through sequential templating approach (STA) to obtain improved catalytic performance by controlling structure and facets (111) of the catalyst [157]. Given that periodically organized metal atom is filled in ZIF-67 (Co) [307], it is noteworthy that utilizing this MOF as a template results in a profound effect on the overall catalytic activity. Since ZIF-67 is formerly constructed from regularly organized Co metal atom (which has a topological resemblance to Co atom in Co_3_O_4_ of HoMSs) and organic ligand, this will assist metal atoms sites and space to associate HoMSs, respectively. Taken together, these provide better charge transfer and improved photocatalytic CO_2_ reduction performance. 

Ultrathin MOFs offer an abundant surface area for photocatalytic reaction with declined diffusion carrier length; this hollow architecture with thin-shell void inside is comparatively favorable for photocatalytic reaction via the provision of the bountiful active site, compacted surface area, and better charge-carrier density [214]. Such architecture could be designed as single- or multi-shell as appropriate to advance performance and reaction conditions [308], and it is viable to assert that this hollow material has a higher catalytic performance to the pristine MOFs [309,310]. In another study, Co-MOF-74 CO_2_ photocatalyst with double-layered-hollow-structured MOF was constructed from the reaction of ZIF-67 to its ligand (MOF-74), 2,5-dihydroxyterephthalic acid (H_4_DOBDC) via solvothermal route, assisted co-catalyst AgNPs@MOF-74 (40 nm-sized Ag-NP incorporated to Co-MOF-74) [311]. Of note, the morphology and structure of Co-MOF-74 have resemblance from the pristine ZIF-67 [312]. 

Although the standalone MOFs photocatalysts play significant role in CO_2_ reduction, the incorporation of co-catalyst evidently increases the photolytic performance kinetically since the role of co-catalyst is to reinforce CO_2_ photolytic process not only by effecting multi-photogenerated electron production and transmission but also it being involved in halting overpotential to manifest the uniformly controlled charge flow within the assembly [313,314,315,316]. As stipulated above, most heterogeneous semiconductors have limited application for the photocatalytic reduction of pollutants due to large bandgap affecting the light absorption spectrum range, while the incorporation of inorganic semiconductor precursor into MOFs has a great impact on performance advancement [317]. Hong et al. reported novel porous heterogeneous co-catalytic material from boron imidazolate framework (BIF-101) crystalline with three-dimensional trinuclear cobalt nodal metal with excellent CO_2_ absorption and reduction capacity, BIF-101, [Co_1.5_(OiPr)_0.5_(OH)_0.5_(H_2_O)(HB(mim)_3_)(NBDC)], where OiPr = isopropyl alcohol [158]. The photoreduction of CO_2_ was accomplished by using BIF-101 in the solvent mixture, Ru-complex PS, yielding 84.1% CO selectivity with H_2_ traces (Table 2). In addition, the stability of BIF-101 against harsh conditions was analyzed and found to be resilient for wider tough reaction conditions (2 < pH < 12). 

Regardless of being great photocatalytic CO_2_ conversion tools due to their uniformly organized structure, large porous available surface area, better surface binding, and ample well-distributed metal activation site, MOFs have low electrical conductivity. This imperfection hinders their standalone application and is a critical challenge for CO_2_ reduction [318,319]. A modified catalyst on MOF’s self-template, targeting photocatalytic CO_2_ reduction capacity enhancement, through appropriate PS coupling with a photocatalyst containing metal with multiple redox states, renders excellent CO_2_ photoreduction by increasing the electrical conductivity, as noted by Xu et al. [160]. 

The electrical properties of MOF have been enhanced via incorporating Co and Te (considering their different electronic configuration) through the tellurization method of precursor ZIF-67 (ZIF = zeolitic imidazolate framework) to fabricate a half-metallic Co_1.11_Te_2_ (with a respective ratio of 1.11:2). Furthermore, carbon coating of this material, Co_1.11_Te_2_⊂C, achieves enhanced BET surface area (107 m^2^·g^−1^) and CO_2_ photocatalytic reduction performance, and the photoreduction process of this heterogeneous catalyst Co_1.11_Te_2_⊂C has been conducted using Ru-based PS, yielding 73.3% CO selectivity over H_2_ within a short time (Table 2); this catalyst outperforms even the pristine precursor material. The outer carbon on this catalyst has been found to be crucial for two reasons: (I) it enhances the photogenerated electron transferability and ionic permeability, and (II) it provides implicit half-metallic character for the heterogeneous catalyst [320]. 

Therefore, the suitable combination of Co and Te [321] on a MOF module would improve the photocatalytic performance of the material not only by increasing conductivity in a minimized manner of charge recombination but also by requiring little free energy for the formation of intermediate substrates in the reduction process.

#### 4.7.5. Graphene-Based Composite MOFs 

The introduction of graphene material into a distinctive framework induces better electrical conductivity and thermal property due to the employment of graphene nanofillers and nanosheets for electronics industries [322,323]. Evidently, the incorporation of graphene nanosheet into the polymeric matrix enhances not only the electronic-structure but also adjusts photothermal stability; moreover, the inclusion of graphene in carbon nanotubes could facilely yield advanced electro-physic-thermal properties [324,325]. This inspires researchers to design graphene material as standalone or along with MOFs for photocatalysts, aiming to achieve better CO_2_ photolytic reduction activity. There are adverse ways for graphene derivatives embodiment to MOFs, supposing the compatibility and band alignment of the host. A 3D structured porous nanosheet composites material is constructed from graphitic carbon (g-C_3_N_4_) and carbon to yield (3D g-C_3_N_4_/C-NPs) material via pyrolysis and carbothermal activation method [326]. 3D g-C_3_N_4_/C-NPs pore volume and the BET surface area have been measured to be 5.825 cm^3^·g^−1^ and 454.2 m^2^·g^−1^, respectively. 

Wang et al. examined the CO_2_ photocatalytic reduction performance of 3D g-C_3_N_4_/C-NPs composite in the presence of water vapor under light illumination, yielding CO (229 µmol·g^−1^) and CH_4_ (112 µmol·g^−1^) [326]. The obtained high reduction capacity of the as-synthesized composite catalyst was attributed to its high gas reactant-product adsorption-sorption morphological virtue, a huge area with enormous activation exposed catalytic site, efficient light-harvesting, and directional transmission of photogenerated exciton with hindered charge recombination. Graphed aggregation with amino-functionalized MOFs exhibited better CO_2_ photolytic performance, to demonstrate this graphene-based MOF (NH_2_-rGO (5 wt.%)/Al-PMOF) for CO_2_ photocatalytic reduction; graphene material possesses high mechanical and chemical stability, large available surface area, high electrical and thermal conductivity, and incorporation into the MOF unlashes superior photolytic performance, with BET surface area of 1180 m^2^·g^−1^ assured to have complete selective CO_2_ reduction to formate (HCOO^−^) potential [143].

## 5. MOFs-Photocatalytic Performance Enrichment 

As formerly discussed, “the assembly of the central metal cluster and organic ligand as a basic building block” is a component-wise definition of all MOFs. Besides the electro-physic-chemical property of this individualistic comprising element, the compatible sensitizer inclusion and synergistic interaction between them has an indisputable impact on the photocatalytic performance of the bundle [327]. As mentioned above, MOFs are a division of porous polymeric material, which could be fabricated in two- or three-dimensional frameworks by using one or more monomers through step-growth or chain growth co-polymerization. Surface modification with metallic NP substantiates the prevalence of additional active catalytic surface area, resulting in the advancement of CO_2_ uptake capacity [328]. Therefore, targeting the obtainment of efficient communication between the nodal metal and organic linker composition and morphological alteration of each component has been a subsistent task. Accordingly, the comprehensive effect of cluster type and/or organic ligand modification on the proper functioning with experimental findings and simulation has been presented afterward. 

### 5.1. Organic Ligand Modification 

The aromatic nature of organic linkers provides structural rigidity to MOF matrices and a longer distance between the binding sites than the aliphatic linkers. Furthermore, these aromatic nature ensures the functionalization of other moieties to boost photolytic activity as a heterogeneous catalyst. Along with amine moiety incorporation to MOFs, metallic NPs embodiment too results in significant improvement in photolytic activity. Along with amine moiety incorporation to MOFs, metallic NPs embodiment too results in significant improvement on photolytic activity. Enlarged BET surface area and enough electron-donating nitrogen sites are required to carry out proper metallic NPs inclusion on MOFs surface [328]. Linkers are liable for solar irradiation harvest and provide photoinduced excitons to the neighboring active catalytic central metal sites to promote adsorbed substrate activation. To assist photon absorption and hot multi-electron injection, this material may be incorporated with other units (organic or inorganic) [91]. An active hydrophobic crystalline MOF (MIL-125-NHCyp, Cyp = cyclopentyl) has been proposed for efficient large-scale photocatalytic reduction of CO_2_ in humid reaction condition without losing its structural integrity [329]. The photoreduction capacities of MOFs based on MIL-125 with different functionalization on aniline in 2-amino-terephthalate were examined for its performance. Three functional groups have been integrated with this base MOF (i.e., -NH_2_, -NHMe, and -NHCyp). The combination, -NHCyp yield MIL-125-NHCyp, has a hydrophobic nature rendered from the steric blocking effect of cyclopentyl group towards hydrogen bond, disallowing bond formation. BET surface areas of MIL-125-NHCyp, MIL-125-NHMe, and MIL-125-NH_2_ before(after) photocatalytic reaction have been measured as 510(503), 1047(1010), and 1473(1411) m^2^·g^−1^, respectively. In comparison, due to the N-alkyl functionalization, MIL-125-NHCyp shows extraordinary structural and crystalline stability (for as long as 20 days) and photocatalytic performance of CO_2_ reduction under humid, flexible acidic/alkali (1 < pH < 9) condition. Furthermore, the as-prepared module retains its structural assembly intact for five redox cycles, as noted by Logan et al. [329].

TiO_2_ is a common semiconductor in solar irradiation harvesting; the incorporation into organic ligand of MOF provides a combined effect of metal-linker functionalization attributable to multi-exciton generation, additional active catalytic site, polarity, porous surface area, and aromatic/lipophilicity [330]. This, in turn, incites overall CO_2_ photocatalytic performance of catalytic matrix. Hybrid MOF has been used for efficient CO_2_ to CH_4_ photoreduction obtained from structurally modified TiO_2_ anatase with phosphoesanoic acid (PHA) functionalization (TiO_2_-PHA) and furnished by porous crystalline shell MOF (HKUST-1) onto growth via step-by-step self-assembly to yield rhombic elongated architecture TiO_2_/HKUST-1 equipped with porous 349 m^2^·g^−1^ surface-area-based N_2_ isotherms [331]. The heterogeneous photocatalytic reduction reaction has been carried out using as-synthesized composite (0.3 g) and water electron donor under visible light irradiation. The enhanced performance of this MOF composite is due to the excellent porous morphological behavior of HKUST-1 and efficient photogenerated exciton transportation from TiO_2_-PHA to HKUST-1 and hole in a reverse way upon irradiation; thereby the redox reaction takes place. Bandgaps of the precursor and composite MOF have been calculated as 3.15, 2.70, and 2.90 eV for TiO_2_-PHA, HKUST-1, and TiO_2_/HKUST-1, respectively, reflecting better photo-responsiveness of a hybrid than TiO_2_ under visible light incident irradiation. 

Moreover, this hybrid exhibits enhanced photocatalytic CO_2_ to CH_4_ with trace CO conversion (yield 1.05 µmol·g^−1^) compared to the pristine constituent (low formation rate of CH_4_ and trace formate). Furthermore, this hybrid has been confirmed to be structurally intact for two rounds. The presence of PHA functionalization in the hybrid MOF plays a significant role in boosting the photolytic performance of the material by (I) effectively improving visible light photoactive TiO_2_ NPs from bare TiO_2_, (II) promoting crystallinity and excited electron penetration to HKUST-1, and (III) owning apt intimate connection-gap integration of hybridization (TiO_2_ and HKUST-1) to avoid agglomeration so as to provide plenty of exposed catalytic active sites for CO_2_ intact reduction. The importance of this functionalizing unit has further been confirmed by its absence in the composite (TiO_2_ + HKUST-1) and results in negligible CO_2_ conversion. Amine functionalization of MOFs shows advanced CO_2_ adsorption-reduction capacity due to the porous topological texture and suitable binding energy for the selective substrate. To elaborate, this is an abundant chemisorption site in MIL-101-Cr through grafting different alkylamine—[i.e., ethylenediamine (EN), diethylenetriamine (DETA), and triethylenetetramine (TETA)]—in the porous surface via post-synthetic modification (PSM) method, using the sulfonic group on the organic linker as an anchor [171]. All the graft PSM, EN-modified material MOF shows excellent photocatalytic activity and durability for room temperature CO_2_ chemisorption. MIL-101-Cr was constructed from terephthalic acid and Cr(NO_3_)_3_·9H_2_O under diluted HF, while MIL-101-SO_3_H was synthesized from 2-sulfoterephthalic acid and concentrated HCl via hydrothermal reaction condition by Xie et al. [332]. 

Then, MIL-101-EN has been synthesized by the immersion of activated MIL-101-SO_3_H in cyclohexane solution of EN by the same procedure, only with the substitution of EN with the respective products denoted as MIL-101-DETA and MIL-101-TETA. N_2_ isotherm indicates that the precursors MIL-101-Cr and MIL-101-SO_3_H have BET surface areas of 3126.5 and 1936.9 m^2^·g^−1^, respectively. Contrastively, the lower CO_2_ absorption capacity of MIL-101-DETA and MIL-101-TETA are attributed to the drastic clogging of surface porosity by DETA and TETA, after the PSM. In the case of MIL-101-EN, EN is introduced into the surface of the MOF in a regulated manner of pore occupancy, resulting in improved photocurrent intensity and advanced long-lived electron transfer with prohibited charge recombination for better chemisorption and reduction potential. 

Therefore, the post-modification of EN-MOF has been confirmed to possess better CO_2_ chemisorption and selective (96.5%, CO) photoreduction compared to the pristine material and other alkylamine derivatives (Table 2) [333] due to its topological and morphological arrangement. Furthermore, this material is durable for four consecutive reaction cycles. 

The peculiar polyoxometalate (POM)-based metal-organic frameworks (POMOFs) [NNU-29] with a hydrophobic ligand group modification are for efficient photocatalytic CO_2_ reduction performance, having higher stability and negligible H_2_ evolution [172]. To fashion this heterogeneous POMOF, reductive nodes of POMs have been examined for better bandgap potential for photolytic preferentiality and Ru-based PS due to more negative CB of the PS (−1.31 V vs. NHE) and photoinduced exciton easily migrating to the catalyst active site [334]. 

**Figure 8:**
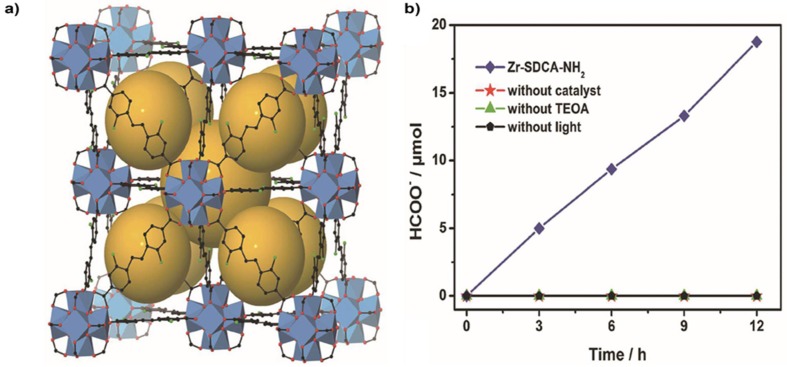
(**a**) 3D network of Zr-SDCA-NH_2_ with void space (yellow spheres), exhibiting two kinds of cages [Color coding (excluding H atom and disordered C and N atoms): Zr(blue), O(red), N(green), and C(black)], (**b**) Yield of HCOO^−^ as a function of irradiation time under altered reaction condition of photocatalyst, sacrificial agent, and light. Reproduced and modified with permission [150]. Copyright 2018, The Royal Society of Chemistry.

The selection of apt functionalization material for the matrices results in the enhancement of environmental substrate adsorption and instant conversion. During the incorporation of the functional material, which increases the electro-physic-chemical structure of the framework, synergistic communication between the assembly components play a fundamental role in reduction performance. Sourced from a large bandgap of the Zr_6_ Oxo cluster, visible-light sensitivity is low, thus the functionalization of the organic linker improves photocatalytic activity. Conjugated amine-functionalization of Zr-based MOFs has confirmed to emerge visible light region active photocatalyst, as reported by Sun et al. [150]. The synthesis of functionalized MOF ([Zr_6_O_4_(OH)_4_(L)_6_]·8DMF, denoted as Zr-SDCA-NH_2_), is influenced by integrating H_2_L1 (H_2_SDCA-NH_2_) in the Zr-framework. The as-prepared photocatalyst has better CO_2_ uptake capacities of 83.8 and 35.2 cm^3^·g^−1^ at 273 and 298 K, respectively. It was noted that the direct proportionality of CO_2_ entropy in accordance with temperature influences absorption capacity. The solvothermal method has been used for the construction of octahedral single-crystal Zr-SDCA-NH_2_ with fcu topological Zr-SDCA-NH_2_ because the planar molecule H_2_SDCA-NH_2_ has a better degree of conjugation and increased light absorption spectrum range (Figure 8a). Due to higher BET surface area, improved physic-chemical stability, and electronic structure, the as-prepared photocatalyst exhibits enhanced substrate photocatalytic performance under sacrificial agent and solution with specified reaction condition producing a formate (HCOO^−^) with a formation rate of 96.2 µml·h^−1^·mmol^−1^ (Table 2), which outperforms other Zr-based MOFs performance in the same reaction condition. This CO_2_ reduction outperformance is attributed to the amine-functionalization of the ligand. To examine the effect of the amine-functional group on catalytic activity, the amine-free ligands (H_2_SDCA) have been used, and the overall light absorption scope is deteriorated (λ = 440 nm). This could be due to the absence of chromophoric amine-functionalized ligand to own enhanced light absorption performance. Having 2.27 eV bandgap, the HOMO and LUMO of Zr-SDCA-NH_2_ were measured to be 1.83 and −0.44 V (vs. NHE) because of more negative CB than the reduction potential of CO_2_ to formate (i.e., −0.28 V vs. NHE), affirming indeed that this MOF is a candidate for CO_2_ photoreduction process. Furthermore, the LUMO of ligand H_2_SDCA-NH_2_ is −0.58 V vs. NHE. This more negative LUMO value implies not only the possible photoexcited electron transfer from the ligand to Zr_6_ Oxo cluster (due to Lewis acidity of Zr^IV^), but also that the organic linker by itself could potentially photo-reduce CO_2_, minding the facilitation of photocatalytic performance via dual organic ligand activity entailed from the amine functionalization. Moreover, the photoreactor ingredients affecting the CO_2_ photolytic conversion were investigated (Figure 8b), demonstrating that without a full set of reaction conditions, the photoreduction mechanism was doomed.

The same Zr^IV^ nodal metal has been scrutinized for efficient photocatalytic CO_2_ reduction through amine functionalization by tunning electronic property [335]. The improved photoelectronic and structural properties of MOF by controlling the composition of (UiO(Zr)-NDI) were fabricated from the NDI-based linker and Zr^IV^ oxide-based cluster. The functionalization of NDI increases the photocatalytic performance of the assembly. Replacement of the NDI linker from outside, inside, and core with amine-moiety has a tremendous effect on the photoinduced charge transfer and photocatalytic performance of modified MOF. Moreover, this geometry optimization could even be achieved by replacing the Zr-metal-in M_6_O_8_ core of the UiO framework by Ti, Th, or Ce. This substitution accounts for the optical bandgap and suitable band edge alignment to boost the photocatalytic performance of the MOF. Employing a functionalization group to the organic ligand framework has shown a marvelous enhancement on overall photocatalytic activity and endurance due to the provision of extra porous surface area and electronic assimilation. Aside from the apropos morphological structure for vicinity substrate adsorption and charge separation of functional group, the extent of synergistic communication with the central metal influences its (moiety) selection and design.

### 5.2. Central Metal Site Modification 

To date, MOFs have drawn the attention of researchers towards environmental remediation, owning enhanced adsorption, abundant active site, various structural adaptabilities, and recyclability [336]. The most common factors affecting the catalytic selective catalysis performance of a given MOF catalyst are structural-coordination, reaction condition, central nodal metal variety [337]. Therefore, the residence of OMSs is used for catalytic activity improvement [338,339]. Atomically scattered photocatalysts containing anchored single metal atoms and/or mononuclear metal complex have been confirmed to be robust to the catalytic activity of the assembly [340].

Doping the central catalytic site of MOFs dispatches the adsorbed substrate activation and transformation instantly upon exciton arrival. The performance of the doped metal cluster activity is greatly influenced by the band alignment, compelling electronic configuration, and dopant activity; therefore, some researches have focused on designing an effective route to nodal metal doping [185]. A stable and photoactive composite MOF of Ru-doped Zr-based MOF (UiO-66) via a templating route to embody Ru-NP onto ZrO_2_ NP of MOF yields efficient peculiar morphological heterogeneous catalyst for photocatalytic reduction of CO_2_ to methanol under visible light illumination, as described by Lippi et al. [341]. The crystallinity nature of as-synthesized MOF is due to precise careful manipulation of metal-oxide and organic components of MOF having better selective substrate reduction performance. 

Central metal clusters are responsible for direct substrate activation, implying that the activity of the nodal metal significantly affects the photolytic performance. Auspicious bimetallic centered MOF (Ni-Co-MOF) for photolytic reduction scheme has been synthesized via microwave-assisted aqueous solution using a BDC linker (benzene-1,4-dicarboxylic acid) for efficient CO_2_ catalytic fixation through CO_2_-epoxide cycloaddition (with epichlorohydrin) [342]. 

Given that water is the solvent for the catalyst synthesis, this process is categorized as environmentally friendly. The as-prepared MOF with 50.8 m^2^·g^−1^ BET surface area and 0.183 cm^3^·g^−1^ pore volume has higher adsorption, selectivity (>99%), and catalytic conversion (96%) performance compared to monometallic analogs (Ni-MOF and Co-MOF). Coordinatively distributed unsaturation of Ni^2+^ and Co^2+^ metal ions on the surface of MOF gives rise to great synergistic intimacy of bi-functionalization assortment. Procedurally, Ni-Co-MOF catalyst effects epoxide and CO_2_ simultaneous activation prior to reaction, thereby lowering the energy barrier of reaction for ignition. Both Ni and Co metal act like Lewis acid sites affecting charge transfer to attack O^−^ of epoxide ring and subsequently affecting the adsorbed CO_2_ to form carbonate complex followed by ring closure. Kurisingal et al. prescribed that Ni-Co-MOF had better reusability (six subsequent rounds) without obvious alteration of morphological and catalytic performance for CO_2_ cycloaddition compared to ZIF-8 or Cu_3_(BTC)_2_ (due to the absence of coordination in respective structure and existence of clogged active site) [343].

Therefore, this novel, cheap, and feasible bimetallic MOF could be a potential tool for CO_2_ fixation. Obviously, in the CO_2_ photocatalytic reduction phenomenon of Zr-based MOF performance, photogenerated electron lifetime plays a significant role. To prolong electrons’ lifetime, the prerequisite energy used to transfer hot excitons from organic linkers to the cluster-based band should be minimum, which, in turn, boosts charge-separation [344]. The prerequisite energy for exciton transfer is termed as energy of ligand to metal charge-transfer (ΔE_LMCT_), and the research on the improvement of UiO-66(Zr) MOF photocatalytic activity has attempted semi- or complete-replacement of Zr^4+^ (d^0^) by Ti^4+^ (d^0^) due to the substitution resulting in lower ΔE_LMCT_ [345,346]. Moreover, the fabrication of MOFs with triple metallic ions would have expanded electron transmission and activation with intact photothermal repression by precluding overpotential. Wang et al. established an isostructural stable MOFs for efficient CO_2_ photocatalytic reduction. Three heterogeneous monometallic photolytic catalysts were designed along with single nodal transition-metal center (Cu^II^, Co^II^, and Ni^II^): MOF-Cu ((Cu_3_(TCA)_2_(dpe)_3_(H_2_O)_3_)_n_), MOF-Co ((Co_3_(TCA)_2_(dpe)_3_(H_2_O)_6_)_n_), and MOF-Ni ((Ni_3_(TCA)_2_(dpe)_3_(H_2_O)_6_)_n_) [173]. 

These three n-type semiconductors have been tested for CO_2_ photocatalytic reduction operation: MOF-Cu, MOF-Co, and MOF-Ni have LUMO values of −1.08, −1.14, and −0.94 V (vs. NHE), respectively, and HOMO values of 0.69, 0.88, and 0.56 eV (vs. NHE), respectively. Bandgaps of MOF-Cu, MOF-Co, and MOF-Ni have been measured to be 1.77, 2.02, and 1.50 eV, respectively. The CO_2_ adsorption capacity of MOF-Cu, MOF-Co, and MOF-Ni has been measured to be 34, 40.35, and 38.87 cm^3^·g^−1^, respectively. Photocatalytic conversion of CO_2_ has been carried out by feeding pure CO_2_ (1 atm and 298 K) in a solvent mixture to the photoreactor pre-loaded with the respective catalyst and [Ru(bpy)_3_]Cl_2_·6H_2_O/[Ru(bpy)_3_]^2+^ PS with TIPA (triisopropanolamine) as sacrificial agent under visible light irradiation. In the same redox reaction condition, these three monometallic MOFs show different yields and selectivities of CO_2_ reduction to CO and H_2_; for instance, the CO_2_ reduction by MOF-Ni yields CO and H_2_ of 22.3 and 0.5 µmol, respectively, while in the case MOF-Cu, the yield of CO declined to 1.7 µmol and H_2_ yield increased to 5.8 µmol. Contrastively, MOF-Ni possesses 98% selective CO evolution (Table 2), indicating the outstanding catalytic performance of the Ni-based monometallic MOF. The reason for this would be better photoinduced exciton charge separation and transmission achieved by not only its lower bandgap (for better incident irradiation harvest and provision of a long-lived electron with retarded charge recombination) but also higher CO_2_ binding energy. 

### 5.3. PS

Articulation of MOFs as an efficient way of pollutant reduction tool is stemmed from their solar energy utilization for activation energy. Despite the generous tailorable physic-chemical structure of MOFs for CO_2_ adsorption, their solar sensibility and conductivity face barriers. Ultimately, designing MOFs with lower bandgap and larger solar absorption spectrum (visible light) is a common target for experimentation. To date, some MOFs barely possess the ability to collect visible light; thus, it is beneficial to advance their solar sensibility by incorporation of PS for further broadening the absorption spectrum. PSs are semiconducting materials designed to assist light irradiation harvest and for exciton provision to the active metal cluster through organic ligand. Therefore, electronic structure (more negative CB, or LUMO) and suitable physical morphology appoints critical effects on the photocatalytic heterostructure performance. Lan et al. reported a 2D MOF from monolayer of MOLsfor PS of photocatalytic CO_2_ reduction; this monolayer 2D material was constructed from Hf_12_-Ru, based on Hf_12_ as a secondary building unit (SBUs) and driven by dicarboxylate ligands [Ru(bpy)_3_]^2+^ (bpy = 2,2′-bipyridine) [180]. 

Both monolayer MOF [Hf_12_-Ru-Re and M(bpy)(CO_3_)X Hf_12_-Ru-Mn] and MOLs’ own [Ru(bpy)_3_]^2+^ PS and M(bpy)(CO_3_)X catalyst for photocatalytic reduction enhancement have been achieved by PSM of carboxylate exchange reaction between Hf_12_-Ru MOL with M(bpy)(CO_3_)X (M/X = Re/Cl and Mn/Br, respectively) moieties. It has been confirmed that the numerous photogenerated electrons from [Ru(bpy)_3_]^2+^ are transmitted to M(bpy)(CO_3_)X. The photocatalytic reduction performance of as-synthesized MOF is obtained by exposing CO_2_ to a catalyst and in TEOA under visible light illumination. From these two, Hf_12_-Ru-Re outperforms the photo-driven CO_2_ reduction, rooted in suitable proximity between MOL scaffolding to the capping, which will facile the electron transfer rate, and, in turn, the photocatalytic reduction reaction of CO_2_.

## 6. Conclusion and Outlook

In recent years, direct CCS techniques have been devised for pollutant remediation; however, eco-friendliness and accessibility of this route entail obstructions. In particular, sorbent corrosiveness and intensive regeneration energy prerequisites remain inexplicable, which stimulates the pursuit of other methods. Thereafter, MOFs evolved: in the assembly of the organic linker and metal cluster, this porous material has the potentiality to adsorb CO_2_ in the environment followed by subsequent photoreduction, mimicking the natural green-plant-photosynthesis carbon cycle process. The mechanism of MOFs CO_2_ photocatalytic reduction is generically externalized into three major subdivisions: (I) CO_2_ adsorption, (II) solar energy collection, and (III) photogenerated exciton generation and conveyance to the metal cluster to execute adsorbed substrate activation and product formation, and desorption of the product thereon. Affordable and environmentally friendly MOFs are manifested with outstanding electro-physic-chemical architecture and design flexibility. Hot effluent gas stream of huge gas- and/or coal-fired power plants contains various components coincidental with CO_2_ (commonly SO_x_, NO_x_, and H_2_O); thus, purification used to be a necessary task for effective choosy trapping in traditional chemisorption. These MOFs are carefully engineered to attain unique inner void-side binding strength range in addition to abundant porous topological texture. This material could be designed in 1D, 2D, or 3D structure, depending on the controlled particle-growth, distribution, and membrane assimilation; moreover, the design of the hollow-cylindrical structure of MOFs has been reported, which has favorable adsorption capacity due to the availability of ample porous surface area. Higher CO_2_ sensibility and adsorption of a given MOF inflicts significantly enhanced photocatalytic performance. 

Moreover, the electronic structure of the MOFs imparts bias on the reduction scope, and the existence of densely OMS and LBS promotes CO_2_ activation. Upon irradiation, the semiconducting unit of the assembly is responsible for effecting hot photogenerated multi-electron injection from the VB, then transmission to the catalytic central metal site in the porous part followed by triggering substrate activation. Solar radiation is abundant; however, classically, it is diffusive with a visible photon density of ~1 photon per nm^2^ per ms, which constricts the maximum photogenerated exciton rate to be not more than ~1 ms^−1^ on PS sole molecule. The fact that most reduction reactions need multiple electrons—for example, CO and CH_4_ evolution reaction, respectively, requires two and eight electrons (Table 1)—and if only one chromophore is installed for photoelectron provision, then apparent side reactions occur during enough electron arrival downtime, which considerably reduces the photocatalytic efficiency of the material. Furthermore, better charge transmission ability of the semiconductor from the exciton site (LUMO) to the catalytic center is an important issue. When the electrons are excited from the VB and head to the CB and ready to depart, leaving a hole behind, if the bandgap energy and incident activation solar energy are unfit, then charge recombination is ensured due to the instant excitation-relaxation phenomenon. Ratifying higher charge collection capacitated material with apt photoelectron-hole separation favors the photoinduced photocatalytic redox reaction. 

Bandgap energy of the junction transistor plays an important role in photon utilization during irradiation; the truncated distance between the VB and CB is committal for smaller activation energy to be used for exciton generation. There are many semiconductors accessible for utilization, but their HOMO-LUMO gap is not suitable for photocatalytic reduction employment (for example, titanium-oxide, TiO_2_). Although TiO_2_ is a cost-effective semiconductor, larger bandgap possession and prevalent charge recombination impede its wider applicability for photocatalytic pollutant reduction, especially if approached as a standalone photocatalyst. Therefore, doping with other metal ions is considered as a countermeasure for bandgap minimization of those semiconductors. Usually, doping a semiconductor with trivalent impurities (such as boron, gallium, and aluminum) results in electron deficiency in the VB, which will act as p-type (acceptor), while if dopant is pentavalent impurities (such as phosphorous), there will be excess electron on the VB, resulting in n-type (donor) semiconductor. Despite their higher electronic sensibility, inorganic semiconductors have lower porosity, entailing smaller CO_2_ adsorption ability. On the other hand, unlike their porous physical structure, the semiconducting feature of MOFs is poor, and the improvement of electronic property typically improves robustness metamorphotic performance. 

Fortunately, viable incorporation of those inorganic semiconductors with MOFs authenticates the electro-photophysical property of the heterostructure enhancement, characterized for provision of long-lived electron for substrate activation and improved uptake amount. As mentioned above, it seems customary to construct MOFs with 1D, 2D, and 3D structures; however, the integrated semiconductors could be in 0D (NPs), but the effect depends on the uniform dispersion of NPs onto the template. 

Composite MOFs outperform the pristine precursor in both adsorption and activation photocatalytic activities because of the concomitant effects of the large-area porous framework and electronic nature of the semiconductor. It is worth noting that the implantation of material to the network improves not only the photoelectronic property but also the gas uptake volume. Ironically, component-compatibility issues are a momentous factor in selecting a material for hybrid formation; band alignment, irradiation responsiveness, lower bandgap, photothermal resilience, proper pore occupancy, better surface area to porous volume ratio, higher CO_2_ affinity, and central metal activity are mentionable aspects. Smooth synergistic interaction between the constituents in the congregated material is necessary for efficient catalytic activity.

Furthermore, the alteration of the nodal metal and organic linker with advanced in-between communication has been asserted to achieve a robust photoreduction process. Central metal could be reinforced/replaced with/by other metal ions, believed to increase the photoelectronic property of the original assembly. Likewise, the inclusion of some metal into the organic ligand will raise the conductivity with passivated charge-hole recombination and adsorption capacity of the strand. The integration of PSs to templet MOFs has been confirmed to be functional in nurturing photon harvest and hot electron injection to the central nodal metal; this n-type semiconductor needs to be protonated from the sacrificial agent upon exciton deportation for decomposition repression and proper-continual operation. In addition, a legitimate assortment of proton donor and semiconductor eventually intensifies the electro-substrate activation route of the compiled heterogeneous photocatalyst in CO_2_ photoreduction. 

Contemplating MOFs as a proficient candidate for photocatalytic CO_2_ reduction, they practically withstand hostile reaction conditions (i.e., humidity, alkalinity, acidity, and heat), which results in expanded appositeness propensity. Frontier MOFs are designed to operate in harsh conditions and have been shown to exhibit intact endurance with lower transformation rate, while others have extraordinary conversion ability, but fragile due to severe condition in-hospitability paves the way for future research. Parallel to the photocatalytic performance of MOFs, replenishment is also a fundamental aspect that needs to be considered to preclude accessibility setbacks; thus, achieving eco-friendly chemical and photothermal accommodation is not a trivial task for MOFs design-fabrication and installation. 

We believe MOFs are efficient tools to mitigate environmental GHG contamination by CO_2_ removal; hence, the discovery of feasible and affordable MOFs should be swift, indicating that these photocatalysts need to be constructed in more photo-sensible and reaction condition resilience mode. Composite MOFs are far more efficient in CO_2_ photoreduction than solo raw MOFs. Material selection is an important factor; MOFs should be designed from great CO_2_-philic porous organic linker to obtain selective substrate adsorption, and this linker needs to be visible-light-sensitive to inject electron to the central metal. Accordingly, exciton collection and substrate activation are central metals’ responsibility, thereby creating higher synergy. It is noteworthy that considering previsouly mentioned features of the organic linker and central metal materials during design have a paramount effect. Apart from building unit selection, fabrication process and installation also significantly affect the catalytic activity. Although there are various MOFs construction processes (such as solvothermal, hydrothermal, and microwave-assisted), process selection depends on the compatibility of the constituent material. Solvothermal and hydrothermal processes are the same, except that the organic solvents are used for solvothermal, while water is used for the hydrothermal process since the chemical reactions in both methods occur in aqueous solution with relatively low temperature (compared to microwave-assisted). We believe that a hydrothermal synthesis method should be the first priority because water is safe to work with and is readily available. More importantly, by this method, it is plausible to control the composition of a crystal during crystalline MOFs’ fabrication. For seeking proper MOFs performance, rating the reaction condition parameters should be carefully devised and controlled upon laboratory simulation. More research needs to be done to fashion MOFs from synergistically harmonious material, for both solo and composite photocatalyst service.

In this review, standalone and composite (two- or three-component) MOFs that work better for CO_2_ photoreduction to renewable hydrocarbon were considered. In each section, we specified the pros and cons of novel innovations. We provided a tabulated data of common MOFs with respective organic linker/cluster and photolytic performance under specified conditions (Table 2). Although the yields were not provided in some of the original work, the persuasive calculation was done for contrastive argument. Moreover, the respective selectivity could be determined by using the given formula. 

## Figures and Tables

**Table 1:** Reduction potential (E) vs. normal hydrogen electrode (NHE) of different intermediate products during metal-organic frameworks (MOF)-driven CO_2_ photolytic reduction coincident with H_2_O oxidation to O_2_.

Reactant Protonation and Electron Inoculation	Product	E (V vs. NHE, at pH = 7)
CO_2_ + 2H^+^ + 2e^−^	HCOOH	−0.61
CO_2_ + 2H^+^ + 2e^−^	CO + H_2_O	−0.53
CO_2_ + 4H^+^ + 4e^−^	HCHO + H_2_O	−0.48
CO_2_ + 6H^+^ + 6e^−^	CH_3_OH + H_2_O	−0.38
CO_2_ + 8H^+^ + 4e^−^	CH_4_ + 2H_2_O	−0.24
2H^+^ + 2e^−^	H_2_	−0.41
H_2_O + 2h^+^	1/2O_2_ + 2H^+^	+0.41

**Table 2:** The photocatalytic performance of MOFs converting CO_2_ to CO, CH_4_, H_2_, and HCOO^−^ with specified reaction conditions.

Photocatalyst	Active Metal Cluster	Organic Metal Linker	BET (m^2^/g)	Photocatalytic Reaction Condition [Catalyst Loading, Solvent, Power, Sacrificial Agent, Irradiation, Time (h)]	Throughput (µmol/g)				Ref.
					CO	CH_4_	H_2_	HCOO^−^	
ZrPP-1-Co	Zr/Co	THPP	852.3	(20 mg), MeCN/TEOA, 300-W xenon lamp (λ > 420 nm), TEOA, 15 h	210	7.5			[139]
Pbz-MOF-1	Zr-O	HCBB	≈1768	(20 mg), MeCN/H_2_O, 300-W xenon arc lamp (λ ≥ 420 nm), TIPA, 12 h				176.5	[140]
PCN-136	Zr-O	HCHC	1768					526	
CsPbBr_3_ QDs (15%)/UiO-66(NH_2_)	Zr-O	H_4_L	465.68	(10 mg), H_2_O/ethyl acetate, 300-W xenon arc lamp (λ ≥ 420 nm), H_2_O, 12 h	98.57	3.08			[141]
MOF-253-Re-(CO)_3_Cl	Re-O	dcbpy		(5 mg), DMF/H_2_O, (400 < λ < 800 nm), TEOA, 4 h	446		22	1900	[142]
NH_2_-rGO (5 wt.%)/Al-PMOF	Zr-O	TCPP	1180	(50 mg), MeCN/TEOA, 125-W mercury lamp, TEOA, 6 h				4113.6	[143]
NH_2_-MIL-101(Fe)	Fe-O	H_4_L		(2 mg), Solvent-Free, 300-W xenon arc lamp (400 < λ < 780 nm), TEOA, 5 h	87.6				[144]
NH_2_-MIL-53(Fe)	Fe-O	H_4_L			15.7				
AUBM-4	Zr-O	cptpy	50	(20 mg), MeCN/TEOA, 150-W (420 < λ < 800 nm), TEOA, 6 h				2196	[145]
PCN-222	Zr-O	TCPP	1728	(20 mg), MeCN/TEOA, 300-W (420 < λ < 800 nm), TEOA, 6 h				2197	[146]
Eu-Ru(phen)_3_-MOF	Eu-O	H_3_L						2205	[147]
NH_2_-MIL-53(Fe)	Fe-O	BDC						2199	[148]
253-Ru(5,5′-dcbpy)(CO)_2_Cl_2_	Ru-O	dcbpy						2200	[149]
MIL-101(Fe)	Fe-O	ATA						2201	[148]
Zr-SDCA-NH_2_	Zr-O	H_2_L1	2546					2202	[150]
NNU-28	Zr-O	H_2_L	1490					2203	[151]
Ir-CP	Ir-ligand	dcbpy		(20 mg), MeCN/TEOA, 500-W (420 < λ < 800 nm), TEOA, 6 h				2204	[152]
NH_2_-UiO-66(Zr)	Zr-O	BPDC	778					2198	[153]
Zr-SDCA-NH_2_	Zr-O	H_2_L1	2546	(40 mg), MeCN/TEOA, 300-W xenon lamp (420 < λ < 800 nm), TEOA, 12 h				470	[150]
co-cat. ZIF-67	Zn-O	TCPP	445	(10 mg), MeCN/MeOH/TEOA, 300-W xenon lamp (420 < λ < 800 nm), TEOA, 6 h	2120		209		[154]
ZnMn_2_O_4_, nanoparticle	Zn-O		2.7	(100 mg), H_2_O, 500-W Xenon arc lamp (λ = 400 nm), TEOA, 8 h	94.7				[155]
ZMO-350	Zn-O	PTCDA	24.7		159.9				
ZMO-450	Zn-O	PTCDA	45.8		191.9				
ZMO-550	Zn-O	PTCDA	109.1		126.6				
ZMO-650	Zn-O	PTCDA	8.4		106.9				
Co_6_-MOF	Co-O	NTB	1957.5	(3 mg), MeCN/H_2_O, 150-W xenon lamp (420 ≤ λ ≤ 780), TEOA,3 h, **PS**	13.120		9376.7		[156]
QS-Co_3_O_4_HoMSs (ZIF-67)	Co-O	2-mIM		(5 mg), H_2_O, 200-W xenon lamp (AM 1.5 filter), 5 h	231.5				[157]
BIF-101	Co-O	NBDC	328.1	(10 mg), MeCN/H_2_O, (λ > 420 nm), 10 h, **PS**	58.300	11.000			[158]
MOF-74	Zn-O	DHBDC		(30 mg), H_2_O, 500-W xenon lamp, 5 h	7.42				[159]
Pt/MOF-74	Zn-O	DHBDC			8.85	9.04			
Au@Pd@MOF-74	Zn-O	DHBDC			12.31				
Pt/Au@Pd@MOF-74	Zn-O	DHBDC			2.42	12.35			
Co_1.11_Te_2_⊂C	Co-/Te-O	2-mIM	107	(1 mg), MeCN/TEOA/H_2_O, 200-W white LEDs lamp, TEOA, 3 h, **PS**	34.200	73.4	12.394		[160]
Ni MOLs (Pure CO_2_)	Ni-O	BDC	48.9	(1 mg), MeCN/TEOA, 5-W white LED light (400 nm ≤ λ ≤ 800 nm), TEOA, 2 h, **PS**	12.500		280		[161]
Ni MOLs (10% dilute CO_2_)	Ni-O	BDC			12.5		0.38		
Co MOLs (Pure CO_2_)	Co-O	BDC	44.6		4.61		2.34		
Co MOLs (10% dilute CO_2_)	Co-O	BDC			0.44		4.15		
Zn_2_GeO_4_			56.4	(20 mg), MeCN/H_2_O, 300-W xenon arc lamp (200 < λ < 1100 nm), 9 h	1.8				[162]
Zn_2_GeO_4_/Mg-MOF-74	Zn-O	H_4_DOBDC	406.7		12.94				
PCN-138	Zr-O	TCPP	1261	(10 mg), MeCN/H_2_O, 300-W xenon lamp (λ ≥ 420 nm), TIPA, 12 h				2021	[163]
Co-cat. Ni_3_(HITP)_2_	Ni-N4/Ni^2+^	HATP	630	(2 mg), MeCN/H_2_O/TEOA, 100-W LED light (λ = 420 nm), TEOA, 3 h, **PS**	103.50		3745		[164]
O-ZnO/UiO-66-NH_2_	Zr/Zn-O	ATA		(100 mg), NaHCO_3_ aq., 300-W xenon lamp (λ > 420 nm), 6 h				29.6	[165]
O-ZnO/rGO/UiO-66-NH_2_	Zr/Zn-O	ATA	877.3					38.5	
TiO_2_			42	(3 mg), 150-W xenon lamp (λ > 325 nm), H_2_, 6 h	17.1				[166]
NH_2_-UiO-66	Zr-O	BPDC	871		9				
1-TiMOF	Zr/Ti-O	BPDC	173		22.44				
2-TiMOF	Zr/Ti-O	BPDC	202		25.44				
3-TiMOF	Zr/Ti-O	BPDC	268		20.22				
4-TiMOF	Zr/Ti-O	BPDC	284		17.1				
MOF-525	Zr-O	TCPP	2127.7	(2 mg), MeCN/TEOA, 300-W xenon arc lamp (400 nm < λ < 800 nm), TEOA, 6 h	384.12	37.2			[167]
MOF-525-Co	Zr-O	TCPP	1317.8		1203.6	220.56			
MOF-525-Zn	Zr-O	TCPP	1264.2		670.2	69.81			
C-Cu_2−x_S@g-C_3_N_4_	Cu-O	TAA	95.1	(1 mg), H_2_O, (300 nm < λ < 800 nm), H_2_O, 12 h	1062.6		26.42		[168]
QS-Co_3_O_4_HoMSs(ZIF-67)	Co-O	2-mIM		(1 mg), H_2_O, 200-W xenon lamp, H_2_O, 5 h	231.5				[157]
g-C_3_N_4_			34.55	(20 mg), MeCN/TEOA, 300-W xenon arc lamp, TEOA, 6 h	32.442	9.294			[169]
BIF-20@g-C_3_N_4_, (10% g-C_3_N_4_)	Zn-O	BH-(mim)^3−^	378.83		170.85	56.1			
BIF-20@g-C_3_N_4_, (15% g-C_3_N_4_)	Zn-O	BH-(mim)3-	283.67		242.75	72.7			
BIF-20@g-C_3_N_4_, (20% g-C_3_N_4_)	Zn-O	BH-(mim)3-	1276.01		305.85	88.15			
BIF-20@g-C_3_N_4_, (25% g-C_3_N_4_)	Zn-O	BH-(mim)3-	1177.77		257	75.35			
AD-MOF-1	Co-O	HAD/BA		(5 mg), MeCN/H_2_O, 300-W xenon lamp (420 < λ < 800 nm), TIPA, 4h				716	[170]
AD-MOF-2	Co-O	HAD/IA		(5 mg), MeCN/H_2_O, 300-W xenon lamp (420 < λ < 800 nm), TIPA, 5 h				1773	
MIL-101-EN	Cr-O	TPA	839.7	(5 mg), H_2_O/TEOA, light-intensity-controlled xenon lamp, TEOA, 10 h	472	17.12			[171]
MIL-101-SO_3_H	Cr-O	TPA	1936.9		217	13			
MIL-101-Cr	Cr-O	TPA	3126.5		83	17			
NNU-29	Zn-O	L		(10 mg), H_2_O/TEOA, 300-W xenon arc lamp (420 < λ < 800 nm), TEOA, 16 h, **PS**	18	58		3520	[172]
MOF-Cu	Cu-O	TCA		(5 mg), MeCN/H_2_O, 300-W xenon lamp (λ ≥ 420 nm), TIPA, 12 h, **PS**	344		1162		[173]
MOF-Co	Co-O	TCA			4564		5062		
MOF-Ni	Ni-O	TCA			4472		104		
TiO_2_			138.07	(10 mg), H_2_O, 300-W xenon lamp (λ > 300 nm), 8 h	6.56				[174]
PCN-224(Cu)	Zr-O	TCPP	2285		29.76	10.86			
15% PCN-224(Cu)/TiO_2_	Zr-O	TCPP	178.05		297.68	1.69			
(Co/Ru)_2.4_-UiO-67(bpydc)	Co-O	bpydc	103.3	(1 mg), MeCN/H_2_O, 450 nm LED light, TEOA, 16 h, **PS**	4520.4		9121.5		[175]
36% CdS/MIL-101(Cr)	Cr-O	TPA	0.1324	(10 mg), H_2_SO_4_ + NaHCO_3,_ (λ ≥ 400 nm), 3.33 h	54.5				[176]
CdS				(10 mg), MeCN/TEOA, 300-W xenon lamp (λ ≥ 420 nm), TEOA, 10 h	230		2480		
CdS/UiO-bpy	Zr-O	bpydc	52.6	(10 mg), MeCN/TEOA, 300-W xenon lamp (λ ≥ 420 nm), TEOA, 10 h			2750		[177]
CdS/UiO-bpy/Co	Zr-O	bpydc	25.5		2350		410		
CsPbBr_3_@ZIF-8	Zn-O	2-mIM		(4.5 mg), H_2_O, 100-W xenon lamp AM 1.5G filter, H_2_O, 3 h	1.515	5.434			[178]
CsPbBr_3_@ZIF-67	Co-O	2-Mim			2.301	10.537			

The data in this table conscript recent MOFs’ CO_2_ conversion capacities in yield with detailed reaction conditions for respective selectivities and references; some data are modified from originally reported units to ensure dimensional homogeneity (µmol per gram of catalyst). **PS** ([Ru(bpy)_3_]Cl_2_·6H_2_O) stands for PSPS, which was discussed in the main body. Selectivity calculation: S = [n_co_/(n_co_ + n_CH4_ + n_H2_ + n_HCOOH_)] × 100%, where n_co_ = yield of CO, n_CH4_ = yield of CH_4_, n_H2_ = yield of H_2_, and n_HCOOH_ = yield of formate.

## Abbreviations/Acronyms

TCPP = tetrakis(4-carboxyphenyl)porphyrine
BH-(mim)^3−^ = boron imidazolate
bpy = 2,2′-bipyridine
dpe = 1,2-di(4-pyridyl)ethylene
bpydc = 2,2′-bipyridine-5,5′-dicarboxylate
cptpy = (4′-(4-carboxyphenyl) -terpyridine)
DHBDC = 2,5-dihydroxybenzene-1,4-dicarboxylic acid
BDC = benzene-1,4-dicarboxylic acid
H_4_DOBDC = 2,5-dioxide-1,4-benzenedicarboxylate
THPP = 5,10,15,20-tetrakis(3,4,5-trihydroxyphenyl) porphyrin
BA = butanedioic Acid
IA = isobutyric Acid
TCBPE = tetrabenzoatetraphenylethylene
ptcda = perylene-3,4,9,10-tetracarboxylic dianhydride
NTB = 4,4′,4″-nitrilotribenzoic acid
NBDC = 2-nitro-1,4-benzenedicarboxylic acid
BPDC = 4,4′-biphenyldicarboxylic acid
TIPA = triisopropanolamine
ATA = 2-aminoterephthalic acid
HCHC = hexakis (4-carboxyphenyl) hexabenzocoronene
HCBB = hexakis(4′-carboxy[1,1′-biphenyl]-4-yl) benzene
TAA = thioacetamide
TCA = 4,4′,4″-nitrilotribenzoic acid ligands
dcbpy = 2,2′-bipyridine-5,5′-dicarboxylic acid
HAD = adenine
TPA = terephthalic acid
BTC = 1,3,5-benzenetricarboxylat
2-mIM = 2-methylimidazole
L = 4,4′-((((perfluoropropane-2,2-diyl)bis(4,1-phenylene))bis(oxy))bis(methylene))dibenzoate anion
H_2_L1 = 2,2′-diamino-4,4′-stilbenedicarboxylic acid
H_2_L = 4,4′-(anthracene-9,10-diylbis(ethyne-2,1-diyl))dibenzoic acid
H_3_L = Ru-L metalloligand
H_4_L = 2-amino-1, 4-benzenedicarboxylic acid (BDC-NH_2_)
H_2_BTTA = 2,5-di(1*H*-1,2,4,-trazol-1-yl)terephthalic acid
NDI = naphthalenediimide
H_2_SDCA = 4,4′-stilbenedicarboxylic acid
MeCN = acetonitrile
H_2_O = water
TEOA = triethanolamine
TIPA = triisopropanolamine
MeOH = metahnol
NaHCO_3_ aqu. = sodium bicarbonate aqueous
BET = Brunauer-Emmett-Teller
TGA = thermogravimetric analysis
TEOA = triethanolamine
TIPA = triisopropanolamine
EN = ethylenediamine
DETA = diethylenetriamine
TETA = triethylenetetramine
ZrPP-1-Co = [Zr_2_(THPP-Co)]_n_
ZIF-8 = Zn(MeIM)_2_
ZIF-67 = Co(MeIM)_2_
UiO-66 = [Zr_6_O_4_(OH)_4_(BDC)_6_]
UiO-67 = [Zr_6_O_4_(OH)_4_(BPDC)_6_]
UiO-bpy = Zr_6_(µ_3_-O)_4_(µ_3_-OH)_4_(µ_1_-OH)(H_2_O)-(bpydc)_5_(HCO_2_)
Ir-CP Y = [Ir(ppy)_2_(dcbpy)]_2_[OH]
HKUST-1 = [Cu_3_(btc)_2_(H_2_O)_3_]
Hf_12_-Ru = Hf_12_(µ_3_-O)_8_(µ_3_-OH)_8_(µ_2_-OH)_6_(TFA)_6_(L-Ru)_6_
L-Ru = bis(2,2′-bipyridine)[5,5′-di(4-carboxyl-phenyl)-2,2′-bipyridine]ruthenium(II) dichloride
BIF-20 = Zn_2_(BH(MeIM)_3_)_2_(OBB)
FJI-H14 = [Cu(BTTA)H_2_O]n·6H_2_O
TCPP-Co = tetrakis(4-carboxyphenyl)porphyrin-Co
PCN-222 = Zr_6_(µ_3_-OH)_8_(OH)_8_-(TCPP)_2_
PCN-136 =(Zr6(μ3-O)4(μ3-OH)4(OH)6(H2O)6(HCHC)
pbz-MOF-1 = (Zr6(μ3-O)6(μ3-OH)2(Ac)5(OH)(H2O)(HCBB))
NH_2_-UiO-66 = [Zr_6_O_4_(OH)_4_(NH_2_-BDC)_6_
MOF-525(Fe) = Zr_6_O_4_(OH)_8_(TCPP-Fe)_2_
MOF-525 = Zr_6_O_4_(OH)_8_(TCPP)_2_
MOF-525-Co = Zr_6_O_4_(OH)_8_(TCPP-Co)_2_
MOF-525(Fe) = Zr_6_O_4_(OH)_8_(TCPP-Fe)_2_
NH_2_-MIL-88B(Fe) = Fe_3_O(solvent)_3_Cl(NH_2_-BDC)_3_(solvent)_m_
NH_2_-MIL-101(Fe) = Fe_3_F(H_2_O)_2_O(NH_2_-BDC)_3_
MOF-253 = [Al(OH)(bpydc)]
MOF-74-Co = [Co_2_(dobdc)]
MOF-5 = [Zn_4_O(BDC)_3_]
MIL-101 = Cr_3_F(H_2_O)_2_O(BDC)_3_·nH_2_O (n ~ 25)
MIL-101(Fe) = Fe_3_F(H_2_O)_2_O(BDC)_3_
MAF-X27-OH = [Co_2_(µ-OH)_2_(bbta)]
MAF-X27-Cl = [Co_2_(µ-Cl)_2_(bbta)]
